# Human papillomavirus, sexually transmitted infections, and antimicrobial resistance in West Africa: Estimating population burden and understanding exposures to accelerate vaccine impact and drive new interventions: The PHASE survey protocol

**DOI:** 10.1371/journal.pone.0332842

**Published:** 2025-09-22

**Authors:** Adedapo Olufemi Bashorun, Larry Kotei, Abdoulie F. Jallow, Ousubie Jawla, Emmanuel U. Richard-Ugwuadu, Muhammed Jagana, Lamin Bah, Amadou Tijan Bah, Karamo Conteh, Mamadou S.K. Jallow, Mehrab Karim, Bai Lamin Dondeh, Anne Segonds-Pichon, Gary M. Clifford, Iacopo Baussano, Bruno Pichon, David Jeffries, Ed Clarke

**Affiliations:** 1 Vaccines and Immunity Theme, Medical Research Council Unit at London School of Hygiene and Tropical Medicine, Fajara, The Gambia; 2 International Agency for Research on Cancer (IARC/WHO), Early Detection, Prevention and Infections Branch, Lyon, France; PLOS: Public Library of Science, UNITED KINGDOM OF GREAT BRITAIN AND NORTHERN IRELAND

## Abstract

Human papillomavirus (HPV) infection is a primary cause of preventable deaths from cervical cancer, a condition of profound inequality with approximately 90% of deaths occurring in low- and middle-income countries, particularly in sub-Saharan Africa. In May 2018, the WHO Director-General declared a Joint Global Commitment to Cervical Cancer Elimination, highlighting the critical role of HPV vaccines in achieving this goal. However, there is a lack of systemically collected data on HPV prevalence in The Gambia, and impact data from high-income countries may not be reliably extrapolated to West African settings due to geographical variation in HPV types and distinct behavioural, biological, and sociodemographic exposures. The Gambia introduced a two-dose HPV vaccination schedule in 2019, but coverage has been very low, interrupted mainly by the COVID-19 pandemic. This presents a key opportunity to generate vital baseline data on HPV prevalence in the population before potential scale-up of vaccination efforts. The PHASE survey, a multi-stage cluster survey, aims to establish the baseline, population prevalence estimates of high-risk and low-risk, vaccine-type and non-vaccine-type HPV infection in 15- to 49-year-old females in The Gambia by measuring urinary HPV-DNA. The survey will also quantify the effects of various exposures on HPV prevalence, including sexual behaviour, the presence of other sexually-transmitted infections (STIs) - Neisseria gonorrhoea (NG), Chlamydia trachomatis (CT), Trichomonas vaginalis (TV), Mycoplasma genitalium (MG), syphilis, as well as blood borne viruses, human immunodeficiency virus (HIV), hepatitis B and hepatitis C; obstetric history, socio-demographic characteristics, and cervical cancer screening and/or treatment. Additionally, the study will provide important antimicrobial resistance (AMR) data for NG and MG in sub-Saharan Africa, a region poorly represented in global surveillance programs. This data is needed to guide regional treatment guidelines and advocate for new solutions, including gonococcal vaccines. The AMR data are expected to immediately influence recommendations regarding the appropriate choice of antibiotics for syndromic STI management in West Africa and hence to address an important driver of AMR in the sub-region. Leveraging on the Medical Research Council Unit The Gambia funded Health Demographic Surveillance system (HDSS) as its sampling frame, the survey will utilize validated diagnostic assays and culturally sensitive data collection methods, to ensure both scientific rigor and local relevance. Tools such as Audio Computer-Assisted Self-Interviewing (ACASI) technology, developed in consultation with local community advisory boards, are included to reduce social desirability bias in reporting sexual behaviour. This approach aims to maximize both the reliability and cultural appropriateness of the findings. This study directly addresses the critical need for baseline epidemiological data on HPV in a West African setting to accelerate vaccine impact and drive new interventions towards cervical cancer elimination. By understanding other factors that influence HPV (like other STIs, sexual behaviour, etc.), the study aims to ensure that, when the vaccine’s impact is measured later, changes in other confounding factors that may impact on HPV prevalence can be accounted for. The study will also establish the population prevalence of the measured STIs and their relationship to common symptoms and other adverse health outcomes related to STIs.

## 1. Introduction

### 1.1. Background

#### 1.1.1. Human papillomavirus and preventable deaths from cervical cancer.

In May 2018, the WHO Director-General declared a Joint Global Commitment to Cervical Cancer Elimination. In the same year, approximately 311,000 women died from cervical cancer worldwide and an estimated 570,000 were newly diagnosed with the condition [1]. Around 90% of the deaths occurred in low- and middle-income countries, and cervical cancer is consistently the first or second most common cause of female cancer-related death in sub-Saharan Africa [1]. Based on current trends, the number of deaths from cervical cancer in Africa are expected to double by 2040, while modelling suggests that Australia, and certain other high-income countries, are on target to reach elimination goals over the same timeframe. Thus, cervical cancer is a condition of profound and increasing inequality.

HPV vaccines will be critical to reversing these trends and ultimately to reaching elimination targets in sub-Saharan Africa. There are currently six licensed HPV vaccines. Cervarix^®^, Cecolin^®^ and Walrinvax^®^ are bivalent vaccines targeting high-risk HPV types 16 and 18. Gardasil^®^ and Cervavac^®^ is are quadrivalent vaccines, additionally incorporating HPV types 6 and 11 which cause anogenital warts, while Gardasil9^®^ is a nonavalent vaccine including a further five high-risk HPV-types (31, 33, 45, 52 and 58) [2,3]. In clinical trials undertaken in high-income countries, these vaccines demonstrated high efficacy against vaccine-type cervical cancer in HPV-naïve 16–26-year-olds [4]. Varying degrees of cross-protection against vaccine-related HPV types have also been reported. Immunobridging studies, based on the comparison of HPV-neutralizing antibody titres, have subsequently been used to support the use in 9–14-year-old females which the WHO recommend be rolled out globally [3,4]. These vaccines were first licensed as a three-dose schedule and later, a two-dose schedule was approved based on immunogenicity and effectiveness data [3]. Currently, a single-dose HPV vaccine schedule has been allowed for by the WHO for most of the HPV vaccines, based on efficacy, effectiveness, and immunogenicity data from several observational studies and clinical trials [3,5–12]. Monitoring the population-level impact of the vaccines in the context of expanding programmatic use in West- and elsewhere in sub-Saharan Africa is now critical. There are no efficacy data from this setting. Furthermore, geographical variation in the prevalence of certain HPV types, as well as distinct, but poorly characterized, behavioural, biological, and socio-demographic exposures in sub-Saharan Africa are likely to influence vaccine impact and make extrapolating data from other settings unreliable. Any impact of pathogen and host-genetic diversity on transmission and persistence is similarly unknown. Meta-analyses of data generated in a limited number of high-income countries indicate that, while the prevalence of vaccine-type HPV has been reduced following vaccine introduction, cross-protection against non-vaccine-type HPV is dependent on achieving high coverage [13]. Similarly, a meta-analysis examining the impact of vaccine introduction on the prevalence of non-vaccine type HPV reported cross-protection against HPV 31, but little or no reduction in HPV 33 and HPV 45, as well as a trend towards an increase in certain high-risk, non-vaccine HPV types [14]. Also, a recent systematic review, reported an increase in HPV vaccine effectiveness, according to the number of vaccine doses received, which would not be predicted based on immunogenicity data from clinical trials alone [15]. Findings from two large reviews of single-dose HPV vaccination schedules indicated that a single dose was highly effective against HPV 16/18, and although the antibody titre levels were stable over the 10-year follow-up periods they were significantly lower than those generated by two- or three-dose HPV vaccine schedule [5,10]. While a single-dose HPV vaccine will significantly simplify logistics, reduce cost, and ease supply constraints, it will be crucial to assess longer-term immunity, potential waning effects, and the context-specific population-level impact of the vaccines following introduction. The reports emphasize the limitations of the data available, even from high-income countries, and the significant risks of bias in current analyses due to the lack of contemporaneously collected data on other exposures. They emphasize the importance of impact studies thoroughly characterising the baseline population prior to vaccine introduction to allow future estimates to be appropriately adjusted [13,15]. In addition, modelling studies have been used extensively to predict the population-level impact of HPV vaccine introduction and have been highly influential in guiding policy [16]. Factors demonstrated to substantially influence estimates of impact include baseline type-specific HPV prevalence and age distribution; individual level vaccine efficacy and age-stratified population coverage; sexual activity patterns including mixing between age groups and sexual activity risk groups; and co-morbidities including malnutrition and other STI. These data further reinforce the fact that impact data from high income countries cannot be assumed to translate to low-income settings in West-Africa, given that differences in many of these factors are likely, but unquantified.

**1.1.1.1. Rationale and impact:** There are no systematically collected data on HPV prevalence in The Gambia. However, in a small study in 20–49-year-old females attending a sexual health clinic in Banjul, the urban capital of the country, HPV 52, which is not included in Gardasil, was the most common high-risk type detected [17]. Only one vaccine-type HPV (HPV 16) was detected amongst the 18 high-risk HPV types reported. In a second study, conducted in a rural setting in The Gambia in 1999, HPV 16 and 18 accounted for 30 of the 93 (32%) of the high-risk HPV types detected in 15–54-year-olds [18]. Establishing sufficiently precise, baseline population estimates of HPV prevalence, and characterizing associated exposures, will allow vaccine impact to be prospectively monitored and confounding minimized. Such data will also enhance predictions from current models, through their context-specific calibration. The dynamic nature of current HPV vaccine policy, as emphasised by the latest WHO Strategic Advisory Group of Experts (SAGE) working group on HPV immunization [19], underscores the critical importance of timely, context-specific data in shaping vaccine policy in West Africa. SAGE has recommended a single-dose schedule in younger women, reflecting a considerable interest in this approach, as well as a two-dose schedule with a 6-month interval in women older than 21 years [19,20]. As of the end of 2024, 20 African countries (excluding The Gambia) have adopted the WHO’s single-dose regimen in their national immunization program [21]. While this schedule offer advantages, in terms of facilitating coverage, and addressing supply and cost constraints, robust data to monitor its impact is essential. Furthermore, with new HPV vaccine manufacturers emerging from developing countries, and their vaccines pre-qualified by WHO, the availability of HPV vaccines is expanding for supply to Gavi-supported countries. While these vaccines also have the potential to overcome supply problems and to reduce cost, ensuring they offer comparable longer-term protection, particularly cross- and herd-protection, given, for example, the use of different adjuvants, will be essential. Modelling studies, to examine the value of higher valency vaccines, and of catch-up vaccinations across wider age cohorts also need context-specific data on HPV types, age distributions, and other exposures from West Africa, for their recommendation to be applied here.

**Fig 1:**
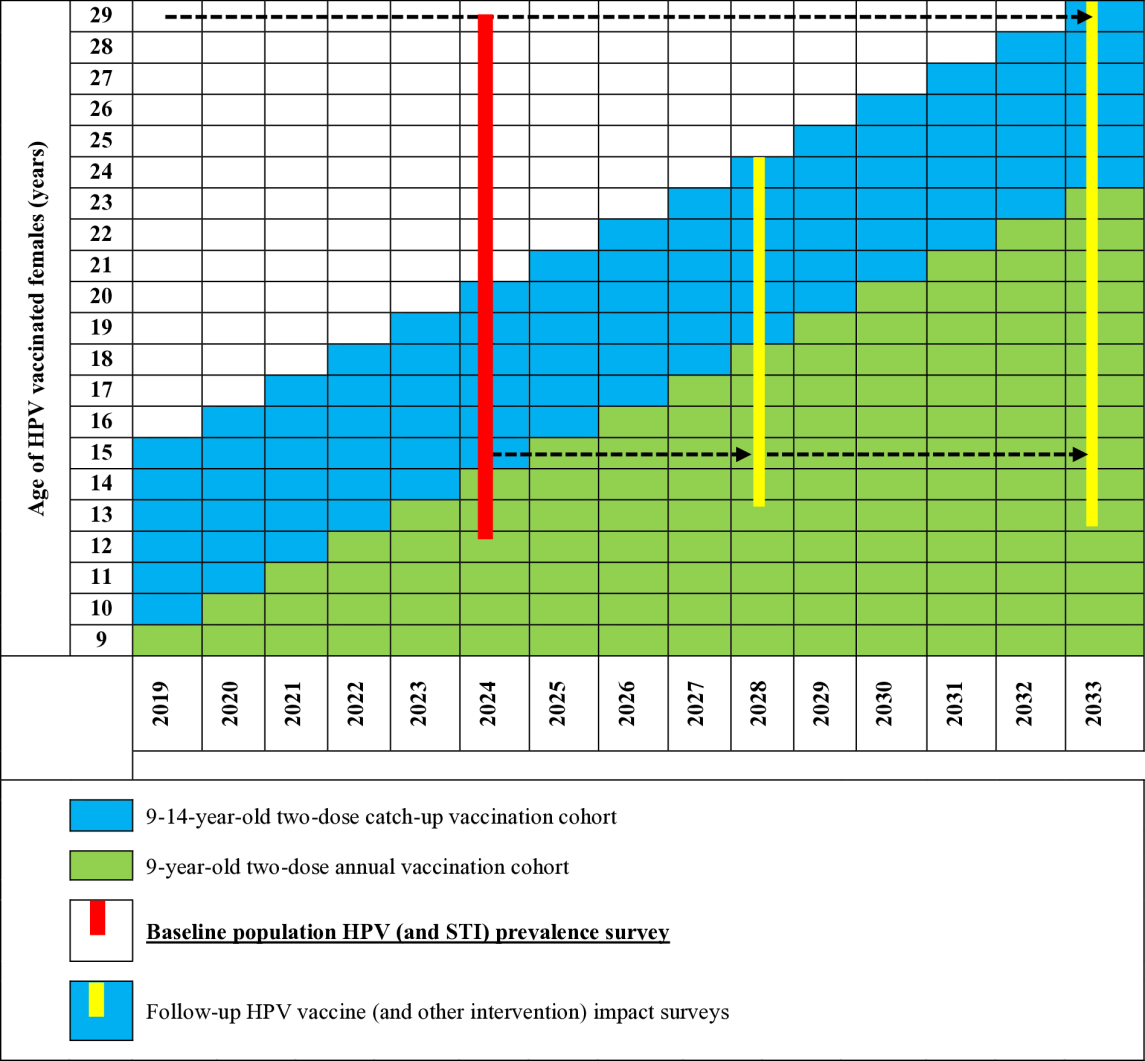
HPV vaccine introduction and future age coverage.

The Gambia introduced a two-dose HPV vaccination schedule to be administered to 9–14-year-old girls in 2019 as a national school-based campaign program [21]. But this program has not been sustained, was largely interrupted by the COVID-19 pandemic and the coverage has been very low, 23% for the first dose in 2024 [21]. This presents a key opportunity to generate these vital baseline data. Fig 1 below illustrates the age of the future HPV-vaccinated female cohort year-on-year.

Additionally, measuring the prevalence of other STIs – NG, CT, TV, MG, syphilis, as well as HIV, hepatitis B and C, will strengthen data quality, enable timely surveillance updates, and improve disaggregated reporting to inform STI and hepatitis programme delivery. These efforts are significant in Western and Central Africa, where service coverage and funding remain limited according to the WHO [22].

#### 1.1.2.. Antimicrobial resistance.

The WHO warns that NG may soon become untreatable due to the global spread of resistance to ceftriaxone and azithromycin, alongside pre-existing resistance to other agents [23]. However, sub-Saharan Africa is underrepresented in WHO-GASP (WHO Global Gonococcal Antimicrobial Surveillance Program), with only 2 of 47 countries submitting AMR data. This gap is critical in West Africa, where syndromic STI management, unregulated antibiotic access, and limited contact tracing likely accelerate resistance. Whole-genome sequencing now reliably predicts AMR in NG and allows mapping of resistant strain spread [24]. The approach also allows high-resolution molecular typing to be undertaken and thus local, regional, and international spread of resistant strains to be mapped [24]. The proposed study will provide the first data of this type from sub-Saharan Africa.

For MG, resistance to azithromycin and emerging resistance to moxifloxacin are growing concerns. Yet, only 72 of 8,966 MG samples included in a recent global AMR meta-analysis were from sub-Saharan Africa—none from West Africa [25].

**1.1.2.1.Rationale and impact:** Findings will directly inform antibiotic recommendations for syndromic STI treatment in West Africa, addressing a key driver of AMR. Moreover, they will support the WHO Global STI Vaccine Roadmap, which calls for better data on NG burden and resistance to model vaccine impact and accelerate development [26].

## 2. Methodology

### 2.1. Study context

The Gambia, a low-income West African country with strong socio-demographic, economic, and cultural links to the sub-region, has a population of 2.4 million, 41% of whom are under 15 years old [27]. Over 90% are Muslim. Early marriage is common: 3.9% of females marry before age 16, and 25.7% before age 19; 34% of women aged 15–49 were in polygamous unions in 2019 [28]. Sexual debut occurs by age 15 in 8.8% of females, by 18 in 43.8%, by 22 in 78.1%, and by 25 in 88.2% [28]. Among 15–24-year-olds, 0.3% of females and 3.3% of males report multiple partners in the past year. Contraceptive use among sexually active, unmarried women is 27.8%, almost exclusively hormonal; barrier use is rare. The total fertility rate is 4.4 children per woman. These figures derive from the 2019–20 Gambia Demographic and Health Survey, based on nationally representative, face-to-face interviews, which may be subject to social desirability bias.

### 2.2. Study objectives

#### 2.2.1. Primary objectives.

- To establish the baseline, population prevalence of high-risk and low-risk, vaccine-type, and non-vaccine type HPV infection through the measurement of urinary HPV-DNA.
- To quantify the effects of the following exposures on HPV prevalence:
- Age at sexual debut; number and age(s) of life-time sexual partner(s); sexual mixing patterns including within and outside polygamous marriages; use of barrier and other contraceptives.
- The presence of other STIs.
- Age at menarche, parity, age at first and subsequent pregnancies, breast feeding.
- Cervical cancer screening and/or treatment.
- Other socio-demographic characteristics (education level, employment, access to water and sanitation facilities, smoking, nutritional status).

#### 2.2.2. Secondary objectives.

- To establish the population prevalence of STIs – Neisseria gonorrhoea (NG), Chlamydia trachomatis (CT), Trichomonas vaginalis (TV), Mycoplasma genitalium (MG), syphilis, human immunodeficiency virus (HIV), hepatitis B and hepatitis C, and their relationship to common symptoms of STIs and other adverse health outcomes related to STIs (e.g., stillbirths, infertility) [29–33].
- To determine the frequency of antimicrobial resistance alleles in NG and MG and associated strain variation

#### 2.2.3. Exploratory objectives.

Exploratory objectives which may be explored within the survey include:

- To determine the seroprevalence of HPV types and other relevant infections (e.g., herpes simplex virus) as a marker of past infection and/or vaccination.
- To establish pathogen and host genetic diversity and their impact on infection prevalence.
- To measure the clustering of HPV types and other STIs, within polygamous marriages and other relevant groups, as a marker of transmissibility.

In examining the clustering of infections within polygamous marriages and other relevant groups, a limited number of males will also be recruited.

### 2.3. Survey outcome measures

#### 2.3.1. Primary.

- HPV types 16, 18, 31, 33, 45, 52, 58, 26, 35, 39, 51, 53, 56, 59, 66, 68, 69, 73, 82 (high-risk types), 6, 11, 40, 42, 43, 44, 54, 61, and 70 (low-risk types) urinary prevalence rates. (*Gardasil® has been used in The Gambia and contains HPV 6, 11, 16 and 18; Gardasil 9® includes HPV 6, 11, 16, 18, 31, 33, 45, 52, 58; Cervarix® contains HPV 16 and 18; Cecolin® included HPV 16 and 18.*)

#### 2.3.2. Secondary.

- NG, CT, TV, MG urinary prevalence rates.
- Syphilis, HIV, hepatitis B and hepatitis C seroprevalence rates.
- Frequency of known AMR alleles in NG and MG.

#### 2.3.3. Exploratory.

- HPV type-specific and additional infection (e.g., herpes simplex virus) seroprevalence rates.
- Pathogen and host genetic diversity as measured by targeted (e.g., human leucocyte antigen typing) or whole genome sequencing (e.g., NG).

### 2.4. Survey design

#### 2.4.1. Overall design summary.

We will conduct a probability-based, multistage cluster survey of females aged 15–49 years using the Basse Health and Demographic Surveillance System as the sampling frame, accounting for clustering at district, village, compound, and household levels. Villages will be selected with probability proportional to size. Following informed consent, data on sociodemographic, obstetrics, sexual mixing, and exposures associated with HPV, other STIs, HIV, and hepatitis B/C will be collected via face-to-face interviews and ACASI in local languages for sensitive topics. First-void urine (Colli-Pee™) will be tested for type-specific HPV, *Neisseria gonorrhoeae*, *Chlamydia trachomatis*, *Trichomonas vaginalis*, and *Mycoplasma genitalium*; HIV, syphilis, and hepatitis B/C will be diagnosed using WHO-prequalified rapid tests. Whole blood will be stored for plasma and DNA. A sub-study of polygamous marriages and other sexual mixing groups will include both sexes to compare HPV/STI transmissibility, with additional targeted sampling as needed to refine estimates.

### 2.5. Participant selection and replacement

#### 2.5.1. Eligibility.

Eligible participants are self-identified females aged 15–49 years, resident in a randomly selected household within the Basse HDSS at the survey visit (defined as having slept there the previous night), able to comply with study procedures, and providing written informed consent/assent. Age will be self-reported or, when available, verified with official documents. This definition includes transient populations. For the sub-study, males in polygamous marriages and other specified groups meeting the same criteria will also be enrolled.

#### 2.5.2. Sampling strategy– population prevalence survey.

The primary aim of the survey is to generate overall estimates of the population prevalence of individual HPV types as well as high- and low-risk, vaccine- and non-vaccine HPV types in 15- to 49-year-olds in the Basse HDSS region. Estimates will be generated for the other STIs, HIV, Hep B and C using the same sampling methodology.

To minimize bias, a probability-based, multistage, random sampling process will be employed. The Basse HDSS will be the sampling frame for the survey. As the sample will be large (>10,000), no age stratification will be necessary, and it is expected that the age distribution will be similar to values in Table 1.

**Table 1:** Age and sex distribution of residents in the Basse HDSS.

Age group (years)	Female N(%)	Male N(%)	Total N(%)
**Less than 15**	44,364 (39.4%)	48,153 (50.5%)	92,517 (44.5%)
**15–19**	12,353 (11.0%)	12,463 (13.1%)	24,816 (11.9%)
**20–24**	11,762 (10.4%)	9,207 (9.6%)	20,969 (10.1%)
**25–29**	8,345 (7.4%)	5,494 (5.8%)	13,839 (6.7%)
**30–39**	14,956 (13.3%)	7,268 (7.6%)	22,224 (10.7%)
**40–49**	9,594 (8.5%)	5,320 (5.6%)	14,914 (7.2%)
**50 & above**	11,258 (10.0%)	7,532 (7.9%)	18,790 (9.0%)
**Total**	112,632	95,437	208,069

#### 2.5.3. Participant recruitment – polygamous marriage and other targeted recruitment.

Participants not selected through the random selection process above and who will not therefore be part of the primary prevalence survey may be approached to take part in the sub-study which aims to better understand the dynamics of transmission in defined sexual mixing groups – predominately polygamous marriages. This will include residents in households, compounds or villages not selected for the prevalence survey. In this case the aim will be to recruit the male (generally the husband) and co-wives. At least two co-wives will be recruited for each male. Sensitisation, consent, as well as data and sample collection and follow-up, will continue in the same way as for the main survey. Given that these individuals are not selected at random in the same way, they will not contribute to the overall survey estimates.

### 2.6. Survey procedures and outcome measure collection

#### 2.6.1. Sensitization.

Selected villages will be visited for community sensitization before the survey, during which a central location (e.g., health post) will be identified for survey activities. Selected households will be approached for individual sensitization with all eligible females (15–49 years), usually in consultation with household heads. Consenting participants will be invited to the central location for data and sample collection.

#### 2.6.2. Informed consent and assent procedures.

Written or thumb-printed informed consent will be obtained from all participants; for those <18 years, parental or guardian consent and participant assent will be required. Consent will be conducted in the participant’s preferred language, verbally translated by trained staff from an approved English script. A literate witness fluent in English will attest to translations. If assent is withheld, minors will not be enrolled regardless of parental views.

**Note:** Only one parent or guardian will be required to provide written consent but in general both parents should agree to participation. The Gambian local languages are not written thus written translation has been proven to be ineffective.

#### 2.6.3. HIV and other pre-test and post-test counselling.

During informed consent, participants will be advised of planned testing. All will receive pre-test counselling from trained staff for HIV and hepatitis B/C in accordance with national and international guidelines. Ongoing verbal consent post-counselling will be documented; those declining testing will be referred to government counselling/testing services. Post-test counselling for HIV and hepatitis B/C will be provided based on point-of-care results, with onward referral as appropriate.

### 2.7. Data collection processes

Data will be obtained via a face-to-face interviewer-administered questionnaire (by trained field staff) and an audio-computer-assisted self-interview (ACASI). Tools will be piloted and adapted based on feedback, without additional approval unless changes have major scientific/ethical implications. Most data will rely on participant recall; when available, official documents (e.g., ID cards, birth certificates, antenatal cards) will be reviewed to improve accuracy, but are not required for participation.

#### 2.7.1. Questionnaire.

Sociodemographic and obstetric data as well as data on family structure and common symptoms of STIs [29–33] will be collected through face-to-face interviews (S1 Appendix) conducted using an online or offline custom-designed REDCap database.

#### 2.7.2. Audio computer-assisted self-interview (ACASI).

Data on sexual history, sexual health, and other topics prone to social desirability bias will be self-administered via ACASI (developed by the survey team on a commercially available software platform provided by SurveyCTO) in Mandinka, Fula, Wolof, and Serahule—spoken but unwritten languages—precluding written English use. Visual prompts of familiar local objects on a tablet touchscreen will guide responses, limited to categorical (including ordered) options due to the inability to enter free text or numerical values. Despite these constraints, ACASI is considered essential for minimizing social desirability bias and enhancing data validity.

### 2.8. Sample collection

#### 2.8.1. First void urine collection.

Participants will self-collect first-void urine using a ColliPee™ device, which captures a defined 20 mL volume into a nucleotide-preserving medium after at least two hours without urination. Residual urine is discarded to the toilet. Collection will follow standardized verbal and pictorial instructions. This method is established for HPV surveys and for detecting NG, CT, MG and TV, though not yet for guiding interventions.

#### 2.8.2. Blood sample collection.

**Fig 2:**
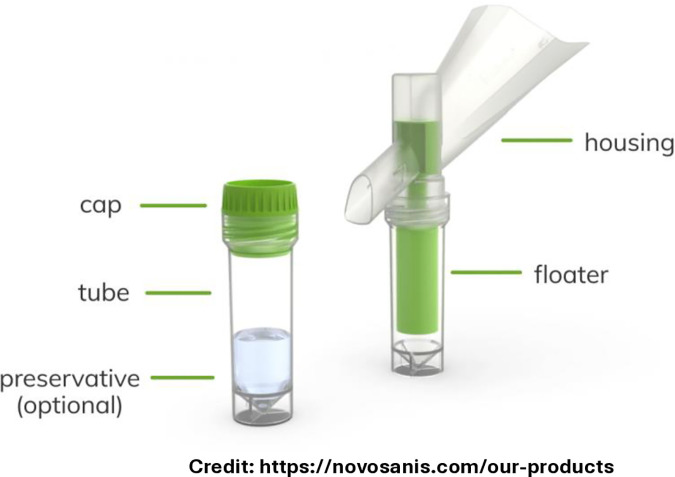
Collipee Device.

3.0mL blood samples will be collected into a clotted tube from all participants by peripheral venepuncture using standard aseptic technique. A small volume of the sample will be retained in the sampling syringe to be used for the designated point of care testing Fig 2.

#### 2.8.3. HPV genital swab collection.

In males recruited to the sub-study examining HPV and STI transmission within polygamous marriages, a saline wetted nylon flocked swab will be collected sampling the penile shaft, glans, foreskin (if applicable) and scrotum [34–36].

#### 2.8.4. Point of care testing.

**2.8.4.1. Pregnancy:** A highly sensitive (detects hCG at a concentration of ≥ 25mIU/mL) urinary pregnancy test, certified against an appropriate regulatory standard, will be undertaken of urine samples obtained fresh in the clinic immediately prior to testing. The manufacturer’s instructions for the performance of the test will be followed.

**2.8.4.2. HIV, hepatitis B and C, syphilis:** WHO-prequalified point-of-care tests (POCTs) will be used to detect HIV-1/2, syphilis, and hepatitis B/C, with sensitivity and specificity >99% and minimal inter-reader variability. Given their diagnostic reliability and WHO-recommended use, no additional laboratory confirmation will be performed; however, plasma samples will be stored for potential later verification.

### 2.9. Laboratory procedures

#### 2.9.1. Urine nucleic acid extraction.

Nucleic acids will be extracted from the first void urine samples using a process optimized to maximise nucleic acid recovery and hence the diagnostic sensitivity of the downstream assays [37–40]. Genomic DNA of HPV, *C. trachomatis*, *N. gonorrhoeae, M. vaginalis* and (*T. vaginalis*), will be extracted from 2 mL of first void urine using the QIAamp DSP mini kit (CAT 61304). Extracts showing high RT-PCR cycle threshold (Ct>=35) will be repeated with twice the volume of urine (4 mL of first void urine).

#### 2.9.2. Urinary HPV laboratory testing.

The Allplex^TM^ HPV28 Detection Assay (cat. no. HP10372X; Seegene) will be used to detect HPV DNA. This real-time PCR assay identifies 28 HPV types – 19 high-risk types (16, 18, 31, 33, 45, 52, 58, 26, 35, 39, 51, 53, 56, 59, 66, 68, 69, 73, and 82) and 9 low-risk types (6, 11, 40, 42, 43, 44, 54, 61, and 70). It covers HPV types included in the vaccines – Gardasil™ (the vaccine introduced in The Gambia [HPV types 6, 11, 16, 18]), Gardasil9™(6, 11, 16, 18, 31, 33, 45, 52, 58), and Cervarix™ (16, 18).

Although the assay is validated for cervical and self-collected vaginal specimens (but not urine), it is currently adopted by the International Agency for Research on Cancer (IARC) for use in HPV prevalence surveys [41]. Its adoption in the PHASE survey ensures comparability with other international surveys. This is further supported by our successful participation in the Global HPV Genotyping Proficiency Study, in which our assay was deemed proficient for detecting HPV types 6, 11, 16, 18, 31, 33, 45, 51, 52, 56, 58, 59, 68a and 68b (S4 Appendix).

Given variable sensitivities and applicability to clinical care, additional assays may also be undertaken for HPV genotyping on samples collected in the survey based on initial results and/or resource availability [42].

#### 2.9.3. Urinary STI testing.

The commercial Allplex™ STI Essential Assay real time PCR (RT-PCR) assay will be used for the detection of CT, NG, MG and TV DNA (SD9801X; https://www.seegene.com/assays/allplex_sti_essential_assay). The assay is suitable for screening for NG, CT, MG and TV on first void urine samples. There are currently no POCT for these four infections which comply with the WHO ASSURED criteria – meeting all requirements of the given target product profiles for these assays [43].

In addition to the 4 four pathogens of interest, STI Essential Assay can detect three additional bacteria DNA: *M. hominis*, *Ureoplasma parvum* and U. *urealyticum.* RT-PCR results will not be reported for clinical care as routine testing and treatment for these pathogens is not recommended in symptomatic or asymptomatic individual by the European STI Guidelines Editorial Board.

#### 2.9.4. Antimicrobial resistance testing.

Alleles known to confer antimicrobial resistance in NG and MG will be identified by RT-PCR based on established protocols [25,44]. Macrolide resistance-associated mutations (MRAMs), predominantly associated with 23S rRNA gene mutations at A2058 and A2059, and quinolone resistance-associated mutations (QRAMs), predominantly associated with *parC* gene mutations (ParC S83 and D87 codons) will be measured [44–46]. Commercially available RT-PCR kits (Seegene Allplex™ MG & MoxiR Assay [SD10233], Allplex™ MG & AziR Assay [SD10170] and Allplex™ NG &DR Assay [SD10368]) will be used for AMR profiling.

#### 2.9.5. Additional exploratory outcome data.

**2.9.5.1. Whole genome sequencing of NG:** Whole-genome sequencing of NG (~100 randomly selected NG-positive samples) will be performed using a newly developed method generating complete genomes from first void urine. Genomic DNA will be extracted from first void urine samples using a customised protocol for host DNA depletion and/or NG DNA enhancement by species-specific DNA baits specifically designed against the 2016 WHO NG reference strains. Genomic DNA will be sequenced using Oxford Nanopore Technology (ONT) for long read sequencing. When necessary, non-NG sequence reads will be filtered out using the NG reference sequence. Dedicated in-house bioinformatics pipelines will be used for AMR determinants detection ([Comprehensive Antibiotic Resistance Database – CARD https://card.mcmaster.ca]; Antimicrobial Resistance [NG-STAR]),), for typing (NG-MAST [www.ng-mast.net], MLST [https://pubmlst.org/], and phylogenetic analyses.

### 2.10. Sample size and sampling design considerations

#### 2.10.1. Sampling frame.

The Basse HDSS (Dec 2023) census was used to define the sampling frame, identifying 59,950 eligible females aged 15–49 across 205 villages (population >30). Data were available at village, compound, and household levels. We assumed sampling coverage of 20%, 40%, and 50% at each respective level, inflating coverage for estimated loss to follow-up of 25%, 30%, and 30%. All eligible female subjects in a household were selected.

To improve geographic balance, the region was divided into four longitudinal tracts:

- West to East: −14.55 to −14.35 (9% of population),
−14.35 to −14.1 (51%),
−14.1 to −13.9 (27%),
• −13.9 (13%).

Villages were selected using weighted simple random sampling (by population size × longitudinal weight), due to poor distribution. A 10,000-iteration simulation was run to minimize imbalance in regional sampling proportions. From the 10 best-balanced outcomes, one was randomly chosen. This process was repeated 1,000 times, yielding 55 final villages. Inclusion probabilities were calculated based on village selection frequency across simulations.

Within selected villages, compounds and households were selected by simple random sampling due to limited population accuracy at finer levels. This process generated a final sampling frame of 21,342 subjects.

#### 2.10.2. Precision simulation.

To assess precision, we simulated 1,000 surveys assuming:

- HPV prevalence of 25%,
- Household-level intra-class correlation coefficient (ICC) of 0.1,
- Coefficient of variation: 20% (between villages), 10% (between compounds), 10% (between households).

**Fig 3:**
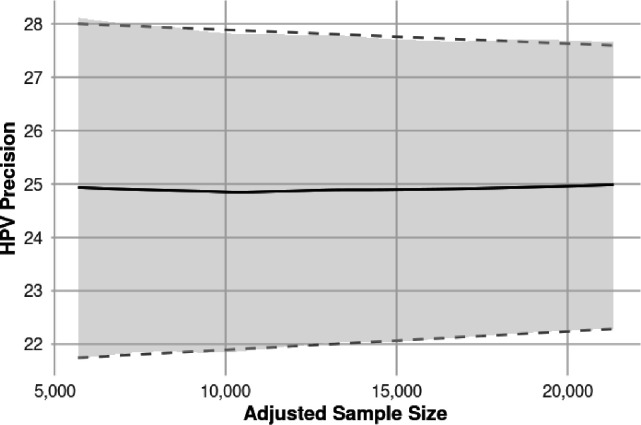
Precision of HPV estimate versus dropouts.

**Fig 4:**
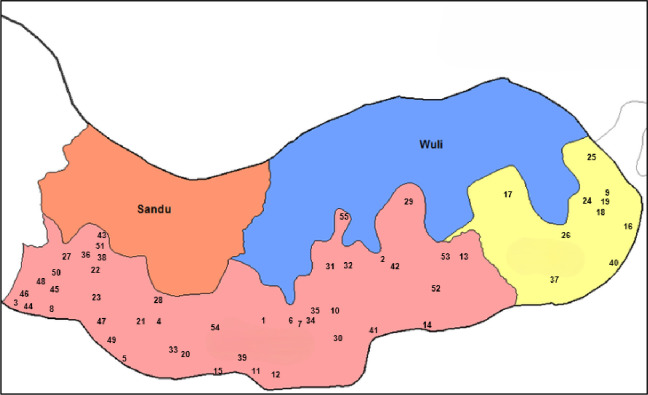
Distribution of the chosen villages in the study area (south Bank of upper river region).

We modelled 11 levels of missingness from worst-case (sample size = 5,686) to full retention (sample size = 21,342), reducing missingness from 100% to 0% in 10% steps. The worst-case scenario assumed 25% of villages, 30% of compounds/households, and 50% of participants were missing. Fig 3 displays 95% confidence intervals for HPV prevalence estimates across these scenarios. Results show precision is stable for 25% prevalence but would be more sensitive for rarer outcomes Fig 4.

#### 2.10.3. Variance estimation and weighting.

Standard errors were estimated using Taylor series linearization to account for the three levels of clustering. Since compounds and households were selected by simple random sampling, their inclusion probabilities were based on the proportion selected at each stage. Village-level inclusion probabilities were derived from the proportion of simulations in which each village was selected.

To address potential bias from design-based weights, which could overemphasize compounds with extreme prevalence and low inclusion probability, a uniform compound-level weight was applied, assuming equal probability for all compounds within a village. This reduced the influence of outliers and improved estimate stability.

Final sampling weights were the inverse of the product of inclusion probabilities at village, compound, and household levels. A finite population correction was applied due to the large sample size.

#### 2.10.4. Participant selection.

Participant selection (sampling frame) will be undertaken by the survey statistician, independent of the field team. The target sample size is a minimum of 10,000 participants, selected based on practical considerations and diminishing returns in precision beyond this threshold, as illustrated in Fig 3. Selected compounds in each village will be surveyed sequentially. To preserve feasibility and avoid over-sampling, no more than 75% of the eligible female population in any village will be surveyed. This will be monitored in real time by the survey team, and recruitment in a village will cease once this threshold is reached.

If 15- to 49-year-old females, who are not listed in the Basse HDSS database, are found to be resident in a household (slept in the household the previous night), they will be included in the survey. This approach helps capture transient populations who may have distinct infection risk profiles.

Villages, compounds, households, and individuals who decline participation will not be replaced. Similarly, missing individuals (non-existent participants due to HDSS inaccuracy, migrated participants, deaths etc) will not be replaced as this has been accounted for in the sampling frame. This pattern of missingness is expected to be consistent across villages. If the target sample size is not met, additional villages will be selected using the same procedure, excluding those initially sampled.

#### 2.10.5. Sub-study of polygamous marriages.

This parallel-group study compares infection clustering within polygamous marriages to higher-level clusters (households, compounds, villages) and between infections, as a proxy for transmissibility/persistence. One arm comprises wives from polygamous marriages; the other, single wives randomly recruited into null clusters. Clusters are limited to sizes 2, 3, or 4 (weighted 10%, 40%, 50% respectively) to reflect population structure. For each arm, intracluster correlation coefficients (ICCs) will be estimated via ANOVA using Smith’s method [47,48], and differences assessed by combining variance estimates to derive 95% CIs [49]. A significant difference is defined as a lower 95% CI > 0. Power was assessed via simulation (≥1,000 iterations), generating designs with specified prevalence, ICCs, and effect sizes; power is the proportion of ICC differences where the lower CI exceeds 0. For example, with a prevalence of 15%, ICCs of 0.02 (compound) and 0.12 (marriage), and 500 clusters per arm, power exceeds 80%.

### 2.11. Statistical analysis plan

**Primary Objectives Analysis:**

- Estimating HPV prevalence: The first primary objective is to estimate the prevalence of HPV infection, categorized by risk (high-risk vs low-risk) and type (vaccine-type vs. non-vaccine-type).
  - Prevalence estimates will be reported for the overall study population and stratified by age group.
  - For each category, point estimates with 95% confidence intervals (CI) will be reported.
  - To account for the multi-level sampling structure (households within compounds, compounds within villages), robust (sandwich) variance estimators will be used to adjust standard errors for intra-cluster correlation. Design effects (DEFFs) will be calculated for each estimate to quantify the impact of clustering on precision (DEFF = variance under clustered sampling/variance under simple random sampling).
- Quantifying the association between exposures and HPV prevalence: The second primary objective is to determine how various exposures affect HPV prevalence.
  - Univariable mixed-effects log-binomial regression analyses will be conducted first to account for the hierarchical clustering of individuals (village> compound> household).
  - ◦ These models will estimate the crude prevalence ratios (PRs) and their 95% CI for each exposure of interest.
  - The exposures include behavioural factors (age at sexual debut, number and age of partners, sexual mixing patterns, contraceptive use), the presence of other STIs, reproductive history (age at menarche, parity, age at pregnancies, breastfeeding), cervical cancer screening and/or treatment, and socio-demographic characteristics (education, employment, access to water and sanitation, smoking, nutritional status).
  - Exposures showing evidence of association (p<0.20) or deemed epidemiologically relevant will be included in multivariable mixed-effects log-binomial regression models, again accounting for clustering.
  - Model selection will be done by backward selection or by epidemiological rationale (e.g., change-in estimate methods).

**Secondary Objective Analysis:**

- Estimating STI prevalence: The secondary objective is to estimate the population prevalence of STIs (NG, CT, TV, MG, syphilis, HIV, hepatitis B, and hepatitis C).
  - Point prevalence estimates with 95% CI will be calculated.
  - Prevalence will be reported for the overall study population and stratified by relevant demographic and behavioural subgroups (e.g., age and each exposure as listed for the primary objectives).
- Assessing associations between STI status, symptoms and adverse outcomes: The associations between STI status and self-reported STI symptoms, as well as selected adverse health outcomes (e.g., stillbirths, infertility), will be assessed.
  - Mixed-effects log-binomial regression models, accounting for clustering, will be used to estimate PRs and corresponding 95% CI.
  - Multivariable models will be selected following the same approach as the primary objectives.
- All analyses will use two-sided p-values with a significance threshold of 0.05, unless otherwise specified. Statistical analysis will be conducted using R and Stata.

## 3. Quality assurance and quality control

### 3.1. Quality assurance

QA procedures will be established for the survey within the existing MRCG Research Governance and Quality Management systems.

#### 3.1.1. Staff and training.

The survey will be conducted by personnel with appropriate qualification, training, and experience to successfully undertake a survey of this nature in The Gambia. Training on all procedures will be undertaken and documented before any survey activities commence.

#### 3.1.2. Standard operating procedures and study specific procedures.

All activities in the survey will be undertaken according to written survey specific procedures (SSP) and/or MRCG standard operating procedures (SOP) as appropriate. SSP outline in detail all aspects of survey conduct, including field, laboratory and data management activities and is written based on the approved protocol. They aim to ensure consistent, high standards of practice across the entire team and serve as reference documents for staff undertaking given procedures. MRCG SOP provide guidance on local ethical and regulatory requirements.

#### 3.1.3. Laboratories and testing.

Point-of-care tests (HIV, hepatitis B and C and syphilis) that have been assessed and prequalified by the WHO will be used throughout. Additional testing (HPV and other STIs) will be carried out using commercially available validated assays established with appropriate controls at MRCG. We successfully participated in the Global HPV Genotyping Proficiency Study, in which our assay was deemed proficient for detecting HPV types (S4 Appendix).

### 3.2. Quality control

QC procedures will be undertaken in real-time throughout the survey, allowing any apparent deficiencies to be addressed at the earliest opportunity.

#### 3.2.1. Data queries.

The eCRF will incorporate on-entry error checks to flag missing, inconsistent, or implausible data in real time for immediate correction. Batch validation checks will be run regularly to ensure cross-field consistency, with queries resolved by field and laboratory teams. Cross-checks will verify alignment between REDCap and ACASI datasets and between recorded and stored biological samples.

## 4. Community and stakeholder engagement

### 4.1. Community Advisory Board (CAB)

A CAB was established to advise on survey design, conduct, and dissemination, particularly given the survey’s sensitive nature. It reviewed all questionnaire items to ensure cultural appropriateness and community relevance. Membership, defined in the CAB Terms of Reference, was selected for broad community representation without language or literacy restrictions. To encourage open communication, female and male members typically meet separately, with joint meetings held when appropriate. The CAB will operate throughout the survey, meeting as needed, and all meetings will be documented.

### 4.2. National Stakeholders

National stakeholders will be engaged during the survey design and throughout survey conduct, and will play an important role in results dissemination. Where treatment is to be provided by the survey team (see Standards of Care), these will aim to align with national guidelines unless there is a clinical reason to deviate. When referral to national medical services, rather than treatment, is appropriate, this will generally be to services provided by or overseen by national stakeholders.

## 5. Ethics/Protection of human participants

### 5.1. Ethical standards

The survey will be conducted in accordance with the Declaration of Helsinki.

### 5.2. Ethics committees

The survey will require the approval of The Gambia Government/MRC Joint Ethics Committee and the London School of Hygiene and Tropical Medicine Research Ethics Committee (S5 Appendix). No survey-related procedures will be undertaken before written approval has been obtained from both committees.

### 5.3. Informed consent process

See section 2.6.2.

### 5.4. Participant confidentiality

Survey data will be stored in a linked, anonymized format using participant ID numbers; no identifiable information will be stored with survey, ACASI, POCT, or laboratory results. Identifiable data (e.g., name, address, contact details) and ID linkage files will be stored separately in locked, access-restricted physical and electronic systems, accessible only to designated investigator team members for operational purposes. ACASI responses will be inaccessible to field teams at the point of collection and viewable only by the data management team (without ID linkage) and senior clinicians.

POCT results will be accessible only to trained testing and counselling staff and survey clinicians; data management staff may view results for validation but without access to identifiers.

### 5.5. Costs and participant compensation

Participants will not be paid to take part in the survey, but will be reimbursed for their time and travel costs (if any) based on MRCG policy on the reimbursement of participants. MRCG holds insurance in the unlikely event that anyone is physically injured due to their participation in the survey.

### 5.6. Standards of care

Participants diagnosed with infections during the survey will receive treatment according to national or international guidelines and/or be referred to appropriate healthcare services. Those unwell or showing signs of complications will be promptly referred to a local facility. The Standard of Care document (S3 Appendix) details referral and treatment protocols. Participants will be encouraged to inform partners or use governmental contact tracing services; however, the survey team will not notify partners, in line with national confidentiality guidance.

#### 5.6.1. Sexually transmitted infections (NG, CT, MG, TV, Syphilis).

Appropriate first-line antibiotic therapy will be provided for participants diagnosed with these STIs. Antibiotic choices will be based on international best practice, adjusted according to local guidelines and/or knowledge of antibiotic resistance patterns when appropriate.

#### 5.6.2. HIV.

Following post-test counselling, participants will be referred on to their preferred HIV treatment centre (which may not be their nearest centre for reasons of confidentiality). When possible and requested, the first clinic attendance will be facilitated by the survey team.

#### 5.6.3. Hepatitis B and C.

Participants testing positive for hepatitis B or C will be reviewed by the MRCG hepatitis clinic to assess disease severity and treatment eligibility. Periodic clinics within the study area, coordinated by the PHASE survey team, will facilitate these assessments. The MRCG team will provide treatment and follow-up as indicated. Pregnant women testing positive for hepatitis B will receive an alert card to support timely newborn hepatitis B vaccination, in line with prevention guidelines. Additional antiviral prophylaxis for preventing mother-to-child transmission is not routinely available in The Gambia.

#### 5.6.4. HPV.

Although HPV testing is established in cervical cancer screening internationally, the validity of first-void urine testing for this purpose remains under evaluation [50–53]. WHO recommends cervical cancer screening from age 30 years, or age 25 years in women living with HIV, due to high spontaneous HPV clearance in younger women [51]. Furthermore, the use of HPV testing as a screening test for cervical disease in women under 30 years in any setting is not recommended based on the high rates of spontaneous viral clearance. Even in those over 30 years of age, HPV screening opportunistically and without rigorous pilot testing of the programme as a whole is not recommended [54]. In addition, Urinary HPV testing, while useful for population estimates (pooled sensitivity 87% [95% CI 78–92%], specificity 94% [95% CI 82–98%]), is not currently recommended for screening purposes [38,55,56].

Health promotion messages during the survey will emphasise accessing available screening services, particularly for participants testing HIV-positive and/or high-risk HPV (HR-HPV).

Following consultation with local experts and the International Agency for Research on Cancer (IARC), all participants aged ≥25 years with HR-HPV will be eligible for screening. This lower-than-WHO age threshold reflects local reports of rising cervical cancer incidence in younger women and was considered to outweigh the potential risk of overtreatment. Scientists at IARC agreed with the decision to lower the screening age in the context of this population-based survey after some deliberation.

Eligibility for cervical cancer screening will be based on the IARC classification of HPV types [57,58]. Specifically, only individuals infected with Group 1 types (HPV 16, 18, 31, 35, 39, 45, 51, 52, 56, 58, 59, and 66) and Group 2A type (HPV 68) will be considered to have HR-HPV types of interest. All other detected HPV types will be considered low-risk and reported as such.

Screening will be performed using visual inspection with acetic acid (VIA) by trained physicians, midwives, and nurses from the SOS Gambia Clinic, who will also provide treatment and follow-up. The PHASE survey team will coordinate screening in batches at accessible locations within the study area. Separate informed consent will be obtained for the use of screening data in further research.

### 5.7. Health promotion and communication activities

Health promotion messages on HPV, STIs, HIV, and hepatitis will be developed in collaboration with the MRCG Communications Department for use during sensitisation and survey activities, aiming to raise awareness of prevention, screening, and treatment services available in The Gambia. A Crisis Communication Plan will be prepared to ensure consistent and coordinated responses to any adverse public communications during the survey.

### 5.8. Future use of stored specimens and data

At consent, participants may choose to allow or decline future use of stored samples beyond the survey objectives, including by the investigator team, external collaborators, or unrelated MRCG researchers. Any such use will require a written application, scientific merit review, and approval by MRCG and The Gambia Government/MRC Joint Ethics Committee, and must be of potential value to the Gambian population. Consent will include permission for possible future genetic testing to maximise sample utility. Samples without future-use consent will be destroyed after survey analyses; otherwise, they will be stored in the MRCG biobank, with retention duration determined by research value. Participants may withdraw consent for future use at any time, after which samples will be destroyed. No identifiable data will be shared, though anonymised demographic or survey data may accompany samples, with negligible risk of re-identification.

## 6. Data handling and record keeping

### 6.1. Data management responsibilities

Data management will be conducted by the MRCG Data Management Department, which will oversee daily operations and provide regular activity reports to the Principal Investigator and study team.

### 6.2. Data capture methods

#### 6.2.1. Data collection in the field.

Questionnaire data will be captured directly into a REDCap™ eCRF, designed with the survey team and data manager, in accordance with GCP and regulatory requirements. REDCap’s offline capability is critical given intermittent internet in the field. ACASI and REDCap data will be integrated for analysis, with ongoing validation to ensure completeness, participant ID alignment, and consistency. Full procedures are outlined in the data management plan.

#### 6.2.2. Laboratory data.

The collection of laboratory samples in the field and the arrival and initial processing and storage of samples in the laboratory will be captured in the REDCap database to allow their easy reconciliation. All samples will be barcoded for storage and the barcodes will also be captured in the database.

Laboratory results on primary and secondary outcomes (HPV types, NG, CT, MG, TV) will be integrated into the REDCap database in blocks as they are generated.

### 6.3. Database development and data validation

The survey database will be developed by the MRCG database developer in line with the Data Management SOP on Database Development, based on investigator requirements. Validation—covering change control, system backup, access control, and security—will be documented before release to ensure compliance with ICH GCP. System and user acceptance testing (UAT) will confirm optimal eCRF flow, correct field formats, functioning validation checks, audit trails, and export functions. Dummy data, including errors, will be used to test both on-entry and batch checks before moving the cleaned system to production.

Data validation—integral to ensuring a complete and accurate dataset for analysis—will use on-entry and batch procedures to identify missing, erroneous, implausible, or inconsistent data. REDCap and ACASI datasets will be cross-checked for participant ID and date alignment, and field sample records reconciled with laboratory logs. All validation and cleaning procedures will be specified in the DMP. Real-time data queries, raised according to predefined error checks, will be resolved through a documented change process with a maintained audit trail. Recurrent errors will prompt targeted or team-wide refresher training.

### 6.4. Timing of report

The data management team will provide weekly reports on key metrics to monitor data quality and promptly address issues. Interim analyses of accrued data, as specified in the survey SAP, may be conducted as needed.

### 6.5. Survey record retention

All records will be retained for a minimum of ten years following the completion of the survey. Decisions to dispose of data will be made by senior MRCG leadership if indicated. The majority of electronic survey data will be retained indefinitely.

## 7. Publication policy

The survey protocol will be published in appropriate peer-reviewed journals to facilitate review and transparency. Data from the survey will be published in open-access peer-reviewed journals based on the reporting procedures defined in the STROBE guidelines [59].

The authorship list will be determined based on the International Committee of Medical Journal Editors (ICMJE) guidelines [60].

## 8. Declarations

### 8.1. Ethics approval and consent to participate

The study was approved by the Gambia Government/MRC Joint Ethics Committee and the LSHTM Ethics Committee. Written informed consent is obtained from all participants, with parental or guardian consent provided for those under 18 years.

### 8.2. Consent for publication

Not applicable.

## 9. Survey status and timelines

The survey field activities started in February 2024, 5,500 participants have been surveyed as of July 2025, and recruitment is expected to be completed by February 2026. Other field activities are projected to end by April 2026, and publication of results by September 2026.[Table pone.0332842.t002]

**Table pone.0332842.t002:** 

SURVEY ACTIVITIES	2022		2023												2024		2025		2026
	N	D	J	F	M	A	M	J	J	A	S	O	N	D	J	F – D	J – O	N – D	J – S
Draft of survey protocol																			
Tour of proposed survey sites																			
Meeting with relevant stakeholders																			
Constitution of the community advisory board																			
Obtaining Scientific and Ethical approval																			
Obtaining approval from the Ministry of Health																			
Develop survey documents and instruments																			
Procurement of survey materials																			
Constituting the survey team and training																			
Pilot survey																			
Community meetings																			
Field survey activities																			
Data cleaning																			
Data analysis and reporting																			

## 10. Discussion

Conducting a large-scale, population-based HPV/STI prevalence survey in The Gambia presented several operational challenges relevant for future surveys in similar contexts. One of the most significant barriers was the timing of recruitment. Fieldwork could not take place during the Muslim fasting period, as questions about sexual behaviour were considered inappropriate during this religious season, particularly in a predominantly Muslim population. Recruitment was also difficult during farming and harvesting seasons, when participants were less available in the mornings. This often resulted in extended working hours for field teams, which contributed to staff fatigue and reduced recruitment efficiency.

Survey activities were further disrupted by local events such as community festivals, political gatherings, and funerals. In some cases, funerals were only communicated to field teams on the day they occurred, requiring suspension of survey activities out of respect for community mourning.

In addition to these seasonal and social constraints, environmental and infrastructural challenges had major impacts. Severe flooding of the MRCG Basse field station and its surrounding area lasted nearly two months, forcing activities to be scaled back to the bare minimum. Vehicle access to the field station was cut off, and staff could only reach the site using a makeshift pedestrian bridge, which significantly hampered logistics, slowed recruitment, and disrupted sample transport and data management.

The design and development of the Audio Computer-Assisted Self-Interview (ACASI) tool—being the first of its kind for a sexual health survey in The Gambia—also required considerable time and resources. This process involved refining culturally appropriate question wording, integrating Community Advisory Board (CAB) feedback, and addressing technical considerations to ensure usability in a low-literacy, low-IT-familiarity population. Community sensitisation meetings further depended on the availability of village heads, whose verbal approval was required before any activities could commence.

Other logistical barriers included delays in procuring essential materials from international suppliers, poor road networks that became nearly impassable during raining season, and extreme temperatures in the Basse Local Government Area, the hottest part of the country, where temperatures often reached 48–50°C. In communities without electricity for fans or air conditioning, these conditions sometimes necessitated early closure of survey sites to protect participants, staff, and laboratory reagents. Sustained mobilisation efforts, including repeated engagement with community leaders, religious figures, and women’s groups, were essential to maintaining recruitment momentum but further extended timelines and increased demands on field staff.

## 11. Strengths and limitations

A major strength of this study was its large, population-based sample, drawn using an age-stratified cluster sampling approach aligned with national demographic patterns, which enhances the generalisability of the findings. The use of ACASI, developed in collaboration with the CAB, was another strength, helping to minimise interviewer bias and support accurate self-reporting of sensitive information. Rigorous laboratory protocols and standardised field procedures further strengthened data quality and reliability.

However, the cross-sectional design limits the ability to establish causal relationships between exposures and HPV/STI infection. While ACASI likely reduced social desirability bias, this influence may still have persisted, especially in a conservative cultural setting. Furthermore, despite intensive community engagement and culturally adapted tool development, some residual cultural barriers may have affected the completeness and accuracy of certain responses.

All authors have read through the manuscript and approved it for submission towards publication in an open-access journal.

## Supporting information

S1 Appendix Face-to-face questionnaire – collecting sociodemographic, obstetrics and gynaecological history, HPV vaccination and some STI data. (PDF)

S2 Appendix ACASI questionnaire – collecting information about sexual behaviour. (PDF)

S3 Appendix Standard of care document. (PDF)

S4 Appendix Global HPV DNA genotyping Proficiency Study 2024 letter – detailing the proficiency of our assay, the Seegene Allplex HPV 28, from the dataset in our study. (PDF)

S5 Appendix Ethical approval letters. (PDF)

## Abbreviations

AMR: Antimicrobial resistance
CT: *Chlamydia trachomatis*
DMP: Data management plan
DNA: Deoxyribonucleic acid
HDSS: Health and demographic surveillance system
Hep B: Hepatitis B
Hep C: Hepatitis C
HIV: Human immunodeficiency virus
HPV: Human papillomavirus
IARC: International Agency for Research on Cancer
MG: *Mycoplasma genitalium*
MRCG: Medical Research Council Unit The Gambia at the London School of Hygiene and Tropical Medicine
NG: *Neisseria gonorrhoea*
PCR: Polymerase chain reaction
POCT: Point of care test
SAP: Statistical Analysis Plan
STI: Sexually transmitted infection
TV: *Trichomonas vaginalis*.
