# Supplementary material for: Human papillomavirus, sexually transmitted infections, and antimicrobial resistance in West Africa: Estimating population burden and understanding exposures to accelerate vaccine impact and drive new interventions: The PHASE survey protocol
Source: PLoS One. 2025 Sep 22;20(9):e0332842. doi: 10.1371/journal.pone.0332842 (PMC12453253; doi:10.1371/journal.pone.0332842)
Supplement: S1 Appendix — (PDF) [file pone.0332842.s001.pdf]

S/N	Variable	Categories	Unit	Remark for the data team
1a	Participant's survey number	XXXXX	counts	5 digits
1b	Participant's code (in capitals)	XX	NA	Only 2 letters
2a	Date of birth is known	Yes	NA	
		No		
2b	Date of birth	DD/MMM/YYYY	Date	Only if q2a is "Yes"
2c	Age	XX	years	min value is 15, max value is 49, only 2 digits allowed
3	Language of interview	Serahule	NA	
		Mandinka		
		Fula		
		Wollof		
		English		
4	How long have you been residing in your household?	XX	unit (days/weeks/months /years)	Only 2 digits allowed. Min value is 01, max value is 10
5	Nationality	Gambian	NA	
		Non-Gambian (please specify)		
6	Ethnicity	Serahule	NA	
		Mandinka		
		Fula		
		Wollof		
		Jola		
		Sere		
		Aku		
		Others (please specify)		the free text - min is 2 letters and max is 15 letters
7	Religion	Islam		
		Christianity		
		Other (please specify)		the free text - min is 2 letters and max is 15 letters

S/N	Variable	Categories	Unit	Remark for the data team
8	Highest educational level attained	None	NA	
		Lower basic (primary) school		
		Upper basic (Junior Secondary) school		
		Senior Secondary School		
		College (post-secondary certificate)		
		Tertiary or University		
		Post graduate		
		Arabic/Quranic studies only		
9	Currently employed?	Yes	NA	
		No		
10	Occupation	Professional/Technica l/Managerial		to disable if q9 is "No"
		Clerical		
		Sales and services		
		Skilled manual		
		Unskilled manual		
		Agriculture		
		Others_specify		the free text - min is 2 letters and max is 15 letters
11a	Type of toilet used?	Flush toilet	NA	
		Pour-flush/covered latrine		
		Open latrine		
		Open defecation		
11b	Is this a private or shared toilet?	Private	NA	Only if 11a is option 1/2/3
		Shared		
12	Type of water source	Private tap/borehole	NA	
		Community tap/borehole		
		Open well		
		Surface/river water		

S/N	Variable	Categories	Unit	Remark for the data team
		Other (please specify)		the free text - min is 2 letters and max is 15 letters
13	Marital status	Single (Never Married)	NA	
		Living together (unmarried)		
		Married		
		Separated		
		Divorced		
		Widowed		
14	Age at first marriage	XX	years	Only if q13 is "Married" or "Separated" or "Divorced" or "Widowed" min value is 12, max value is 49
15	Number of marriages	XX	counts	Only if q13 is "Married" or "Separated" or "Divorced" or "Widowed" min value is 01, max value is 10
16	Are you in a polygamous or monogamous marriage?	Monogamous	NA	Only if q13 is "Married"
		Polygamous		
17	How many wives husband has?	XX	counts	Only if q14 is "Polygamous" min value is 01, max value is 04
18a	Have you attained menarche?	Yes		
		No		
18b	Age at menarche	XX	years	Only if q18a is "Yes" min value 08, max value 25
19a	Have you ever been pregnant?	Yes		Only if q18a is "Yes"
		No		

S/N	Variable	Categories	Unit	Remark for the data team
19b	Age at first pregnancy	XX	years	Only if q19a is "Yes", min value 12, max value 49
20	Number of pregnancies	XX	counts	Only if q19a is "Yes", min value 01, max value 30
21a	Number of live births	XX	counts	Only if q19a is "Yes", min value 00, max value 30
21b	Number of child(ren) alive?	XX	counts	Only if q21a is ≥01 min value 00, max value 30
22a	Have you ever had a pregnancy that ended in stillbirth? i.e. pregnancy ≥20 weeks	Yes	NA	Only if q19a is "Yes"
		No		
		Prefer not to answer		
22b	If yes, how many?	XX	counts	Only if q19a & q22a is "Yes" min value 01, max value 10
23a	Have you ever had a pregnancy that ended in miscarriage? i.e. pregnancy <20 weeks	Yes	NA	Only if q19a is "Yes"
		No		
		Prefer not to answer		
23b	Can you tell us how many miscarriages you have had?	XX	counts	Only if q19a & q23a is "Yes" min value 01, max value 10
24	Age of oldest child	XX	unit (days/weeks/months /years)	Only if q21b is ≥01 min value is 01, max value should not be greater than age of participant
25	Age of younger child	XX	unit (days/weeks/months /years)	Only if q21b is >01 min value is 01, max value should not be greater than age of participant

S/N	Variable	Categories	Unit	Remark for the data team
26	Did you breastfeed your children?	Yes		Only if q21a is ≥01
		No		
27	Are you currently using or have used any method to delay or avoid getting pregnant?	Yes, currently using	NA	
		No		
		Yes, but not using any currently		
28a	What types of method to delay or avoid pregnancy are you using now?	Injectable/implant		Only if q27 is "Yes, currently using"
		OCP/Pill		
		condom/barrier		
		intrauterine device		
		surgical sterilization - Male or female		
		Lactational amenorrhoea method		
		Periodic abstinence		
		withdrawal method		
		Traditional/herbal methods		
		Others_specify		the free text - min is 2 letters and max is 15 letters
28b	Can you estimate how long you have been using the method for?	XX	unit (days/weeks/months /years)	Only if q27 is "Yes
29a	What type of method to delay or avoid pregnancy did you use before?	Injectable/implant	NA	Only if q27 is "Yes, but not using any currently"
		OCP/Pill		
	**(choose all that apply)**	condom/barrier		
		intrauterine device		

S/N	Variable	Categories	Unit	Remark for the data team
		surgical sterilization		
		Lactational amenorrhoea method		
		Periodic abstinence		
		Withdrawal method		
		Traditional/herbal methods		
		Others_specify		
29b	Can you estimate how long you used the method(s) for?	XX	unit (days/weeks/months /years)	Only if q27 is "Yes, but not using any currently", min value is 01, max value is 10
30a	Do you drink alcohol?	Yes		
		No		
30b	In the past year, how often did you drink any alcoholic beverage? E.g. beer, wine, spirit or locally brewed beer	every day	NA	If 30a is "Yes"
		1 to 4 times weekly		
		Few times a year		
		Occasionally		
31	Do you smoke cigarette?	Yes	NA	
		No		
32a	Have you ever received a vaccination against HPV?	Yes	NA	
		No		
		Not sure		
32b	Did you ever receive an HPV vaccination card?	Yes	NA	Only if q32a is "Yes"
		No		
		Not sure		

S/N	Variable	Categories	Unit	Remark for the data team
33a	How many doses of HPV vaccines have you received?	X	counts	Only 1 digit allowed, min value is 0, max value is 3
33b	Which year did you receive your first HPV vaccine dose?	YYYY	year	Only if q32 is "Yes" min value is 1999 max value is the current year
34	Have you heard of cervical cancer screening?	Yes	NA	
		No		
35	Do you know what cervical cancer screening involves	Yes (correctly described)		
		No (incorrectly described)		
36	Previous screening for cervical cancer?	Yes	NA	
		No		
		Not sure		
37	Have you been treated for cervical cancer/pre-cancer (in the hospital/clinic)	Yes		Only if q34 is "Yes"
		No		
		Not sure		
38a	Age of husband	Older than me	NA	Only if q13 is "Married"
		About the same age		
		Younger than me		
38b	Where does your husband live?	Under the same roof/same household		Only if q13 is "Married"
		Same compound		
		Same village, different compound		
		Nearby village		
		Another part of The Gambia		
		Outside the country		
39a	Is your husband currently working?	Yes	NA	Only if q13 is "Married"
		No		

S/N	Variable	Categories	Unit	Remark for the data team
		**Not sure**		
39b	What work does your husband do?	Professional/Technical/Managerial	NA	Only if q13 is "Married" & q39a is "Yes"
		Clerical		
		Sales and services		
		Skilled manual		
		Unskilled manual		
		Agriculture		
		Not sure		
		Others_specify		
40a	Age of partner	Older than me	NA	Only if q13 is "Living together (unmarried")
		About the same age		
		Younger than me		
40b	Where does your partner live?	Under the same roof/same household	NA	Only if q13 is "Living together (unmarried")
		Same compound		
		Same village, different compound		
		Nearby village		
		Another part of The Gambia		
		Outside the country		
41a	Is your partner currently working?	Yes	NA	Only if q13 is "Living together (unmarried")
		No		
		**Not sure**		
41b	What work does your partner do?	Professional/Technical/Managerial	NA	Only if q13 is "Living together (unmarried") & q41a is "Yes"
		Clerical		

S/N	Variable	Categories	Unit	Remark for the data team
		Sales and services		
		Skilled manual		
		Unskilled manual		
		Agriculture		
		Not sure		
		Others_specify		
	**STI related questions**			
42	Have you heard about sexually transmitted infections before apart from this study?	Yes	NA	
		No		
		Not sure		
43	Where did you hear or learn about sexually transmitted infections? (choose all that apply)	TV		Only if q42 is "Yes"
		Radio		
		school		
		Health centre or hospital		
		Family or friends		
		community meeting		
		Social media - WhatsApp, Facebook, instagram etc		
		Other (please specify)		
44	How can you protect yourself from sexually transmitted infections? (choose all that apply)	Use of a condom		Only if q42 is "Yes"
		Not having sex		
		Not having multiple sexual partners		
		vaccination		
		prayers		
		testing and treatment for STIs		

S/N	Variable	Categories	Unit	Remark for the data team
		Herbal concoction		
		local ways or traditions		
45	Have you had any infection which you got through having sexual intercourse?	Yes	NA	
		No		
		Not sure		
46	The last time you had any form of STI did you seek treatment?	Yes	NA	Only if q45 is "Yes"
		No		
47	From where did you first seek treatment?	Health centre		Only if q45 is "Yes"
		Pharmacy		
		Local treatment		
		Religious centre		
		Friend, relative		
		Other (please specify)		
48	ID of staff completing the form	XXX		3 digits
