# Supplementary material for: Human papillomavirus, sexually transmitted infections, and antimicrobial resistance in West Africa: Estimating population burden and understanding exposures to accelerate vaccine impact and drive new interventions: The PHASE survey protocol
Source: PLoS One. 2025 Sep 22;20(9):e0332842. doi: 10.1371/journal.pone.0332842 (PMC12453253; doi:10.1371/journal.pone.0332842)
Supplement: S2 Appendix — (PDF) [file pone.0332842.s002.pdf]

S/N	Variable	Type	Categories	Unit	Constraints
1a	When was the last time you had sexual intercourse?	Categorical	Today		**only for those that have never been pregnant**
			1 to 3 days		
			In the last week		
			Within the past 4 weeks		
			Within 1 year		
			More than 1 year ago		
			Never had sexual intercourse		
			Prefer not to answer/cannot remember		
1b	When was the last time you had sexual intercourse?	Categorical	Today		**only for those that have been pregnant**
			1 to 3 days		
			In the last week		
			Within the past 4 weeks		
			Within 1 year		
			More than 1 year ago		
			Prefer not to answer/cannot remember		
2	How old were you when you first had sexual intercourse?	both	less than 12 years		**only if q1a/b is option "1 - 6" or q1a/b=prefer not to answer/cannot remember**
			12 to 14 years		
			15 to 17 years		
			18 to 20 years		
			More than 20 years		
			Prefer not to answer/cannot remember		

S/N	Variable	Type	Categories	Unit	Constraints
3	Who was the person you first had sexual intercourse with?	Categorical	Someone from outside the village/community that you knew		**only if q1a/b is option "1 - 6" or q1a/b=prefer not to answer/cannot remember**
			Someone from outside the village/community that you just met		
			Someone in the same village/community who was not your husband/boyfriend		
			Long term partner to whom you were not married to		
			Husband		
			Prefer not to answer/cannot remember		
4	Can you tell us which of the following is true?	Categorical	In the last 1 year, you have had sexual intercourse with only 1 man	NA	**only if q1a/b is option "1 - 5" or q1a/b=prefer not to answer/cannot remember**
			In the last 1 year, you have had sexual intercourse with 2 men		
			In the last 1 year, you have had sexual intercourse with 3 men		
			In the last 1 year, you have had sexual intercourse with 4 men		
			In the last 1 year, you have had sexual intercourse with 5 to 10 men		
			In the last 1 year, you have had sexual intercourse with more than 10 men		
			Prefer not to answer/cannot remember		
5	Of all the men you've had sexual intercourse with, can you describe their ages?	Categorical	Always older than you	NA	**only if q1a/b is option "1 - 6" or q1a/b=prefer not to answer/cannot remember**
			Mostly older than you		
			About the same age		

S/N	Variable	Type	Categories	Unit	Constraints
			Mostly younger than you		
			Always younger than you		
			Prefer not to answer/cannot remember		
6	Indicate the men you ever have had sexual intercourse with? (Choose all that apply)	Categorical	Someone from outside the village/community that you knew	NA	**only if q1a/b is option "1 - 6" or q1a/b=prefer not to answer/cannot remember**
			Someone from outside the village/community that you just met		
			Someone in the same village/community who was not your husband/boyfriend		
			Long term partner to whom you were not married to		
			Husband		
			Prefer not to answer/cannot remember		
7	Has any man forcefully had sexual intercourse with you before (against your will)	Categorical	Never	NA	**only if q1a/b is option "1 - 6" or q1a/b=prefer not to answer/cannot remember**
			A few times		
			Many times		
			Prefer not to answer/cannot remember		
8	Have you ever had sexual intercourse or been sexually involved with anyone because they gave you or told you they would give you material support like gifts, money, rent, school fees or anything else?	Categorical	Never	NA	**only if q1a/b is option "1 - 6" or q1a/b=prefer not to answer/cannot remember**
			A few times		
			Many times		
			Prefer not to answer/cannot remember		

S/N	Variable	Type	Categories	Unit	Constraints
9	Have you ever provided material support like money/gifts etc. to your male partner in appreciation for sexual intercourse?	Categorical	Never		**only if q1a/b is option "1 - 6" or q1a/b=prefer not to answer/cannot remember**
			A few times		
			Many times		
			Prefer not to answer/cannot remember		
10	Have you ever had any sexual intercourse with another man apart from your husband while still married to him?	Categorical	Never	NA	**only if q1a/b is option "1 - 6"**
			A few times		**or if once married or q1a/b=prefer not to answer/cannot remember**
			Many times		
			Prefer not to answer/cannot remember		
11	Of all the sexual intercourse you have had in the last 12 months, how often has your male partner used a condom?	Categorical	Never		**only if q1a/b is option "1 - 5" or q1a/b=prefer not to answer/cannot remember**
			A few times		
			Many times		
			Always		
			Prefer not to answer/cannot remember		
12	In the last 3 months, have you had a new sexual partner?	Categorical	Yes		**only if q1a/b is option "1 - 5" or q1a/b=prefer not to answer/cannot remember**
			No		
			Prefer not to answer/cannot remember		
13	Have you ever had anal sex?	Categorical	Never		
	Anal sex is when the male put his penis in the female's anus for sexual pleasure.		A few times		

S/N	Variable	Type	Categories	Unit	Constraints
			Many times		
			Prefer not to answer/cannot remember		
14	Have you ever had oral sex?	Categorical	Never		
	Oral sex is when the male put his penis in the female's mouth or the female licks or sucks his penis, or he licked/sucked the female's vagina		A few times		
			Many times		
			Prefer not to answer/cannot remember		
15	Have you ever engaged in having sexual pleasure with any other woman before?	Categorical	Never		
			A few times		
			Many times		
			Prefer not to answer/cannot remember		
16	Have you ever engaged in genital touching?	Categorical	Never		
	Genital touching when the female touches the male partner's penis with her hand, he touches her vagina with his hand or he rubbed his penis on her legs/buttocks/genitals but did not have sexual intercourse		A few times		
			Many times		
			Prefer not to answer/cannot remember		
17	Do you clean inside of your vagina?	Categorical	Never		
	Vaginal cleansing is cleaning inside the vagina with water, soap or other products using fingers or a cloth or sponge		A few times		
			Many times		
			Prefer not to answer/cannot remember		
18	During your menstrual period, which of the following best describes what you use?	Categorical	Menstrual Pad		
	(Multiple choice)		Cloth		
			Tissue		
			Pant only		

S/N	Variable	Type	Categories	Unit	Constraints
			Not experienced my first period yet		
19	There are some women who have had female genital cutting before. Have you ever had this done on you?	Categorical	Yes	NA	
			No		
			Prefer not to answer/cannot remember		
20	Was your most recent male sexual partner circumcised?	Categorical	Yes	NA	**only if q1a/bis option "1 - 6" or q1a/b=prefer not to answer/cannot remember**
			No		
			Prefer not to answer/cannot remember		
21a	In total, how many men have you ever had sexual intercourse with?	Categorical	1	NA	**only if q4 is option "1"**
			2		
			3		
			4		
			5 to 10		
			> 10		
			Prefer not to answer/cannot remember		
21b	In total, how many men have you ever had sexual intercourse with?	Categorical	2		**only if q4 is option "2"**
			3		
			4		
			5 to 10		
			> 10		
			Prefer not to answer/cannot remember		
21c	In total, how many men have you ever had sexual intercourse with?	Categorical	3		**only if q4 is option "3"**
			4		

S/N	Variable	Type	Categories	Unit	Constraints
			5 to 10		
			> 10		
			Prefer not to answer/cannot remember		
21d	In total, how many men have you ever had sexual intercourse with?	Categorical	4		**only if q4 is option "4"**
			5 to 10		
			> 10		
			Prefer not to answer/cannot remember		
21e	In total, how many men have you ever had sexual intercourse with?	Categorical	5 to 10		**only if q4 is option "5"**
			> 10		
			Prefer not to answer/cannot remember		
21f	In total, how many men have you ever had sexual intercourse with?	Categorical	> 10		**only if q4 is option "6"**
			Prefer not to answer/cannot remember		
	**SEXUAL BEHAVIOURAL QUESTIONS (HUSBAND OR MALE PARTNER)**				
22	Do you know if your husband has had any STI in the last 12 months?		Yes	NA	**for only those who are married**
			No		
			Not sure		
23	Do you know if your husband has sexual intercourse with another woman apart from you or co-wife?	Categorical	Yes	NA	**for only those who are married**
			No		
			Not sure		
24	Do you know if your husband has ever had sexual intercourse with another man?	Categorical	Yes	NA	**for only those who are married**
			No		
			Not sure		

S/N	Variable	Type	Categories	Unit	Constraints
25	Do you know if your boyfriend has had any STI in the last 12 months?	Categorical	Yes	NA	
			No		
			Not sure		
			I do not have a boyfriend		
26	Do you know if your boyfriend is having sexual intercourse with another woman apart from you?	Categorical	Yes	NA	
			No		
			Not sure		
			I do not have a boyfriend		
27	Do you know if your boyfriend has ever had sexual intercourse with another man?	Categorical	Yes	NA	
			No		
			Not sure		
			I do not have a boyfriend		
