# Supplementary material for: Human papillomavirus, sexually transmitted infections, and antimicrobial resistance in West Africa: Estimating population burden and understanding exposures to accelerate vaccine impact and drive new interventions: The PHASE survey protocol
Source: PLoS One. 2025 Sep 22;20(9):e0332842. doi: 10.1371/journal.pone.0332842 (PMC12453253; doi:10.1371/journal.pone.0332842)
Supplement: S3 Appendix — (PDF) [file pone.0332842.s003.pdf]

ABBREVIATIONS, DEFINITIONS AND CONTRACTIONS

- AFP – Alpha-fetoprotein
- AIDS – Acquired Immune Deficiency Syndrome
- ALT – Alanine aminotransferase
- ARV – Antiretroviral
- ART – Antiretroviral therapy
- AST – Aspartate aminotransferase
- CDC – Centers for Disease Control and Prevention
- CT – Chlamydia trachomatis
- DAA – Direct-acting antiviral
- DNA – Deoxyribonucleic acid
- EASL – European Association for the Study of the Liver
- HBsAg – Hepatitis B surface antigen
- HBV – Hepatitis B virus
- HCV Ab – Hepatitis C virus antibody
- HBeAg – Hepatitis B e-antigen
- HIV – Human Immunodeficiency Virus
- HPV – Human papillomavirus

- IM – Intramuscular
- IU – International units
- MRCG – Medical Research Council Unit The Gambia at the London School of Hygiene and Tropical Medicine
- MG – Mycoplasma genitalium
- NG – Neisseria gonorrhoeae
- PHASE – Prevalence of HPV, Antimicrobial resistance, and STI Estimation
- POC – Point of care
- RNA – Ribonucleic acid
- SCJ – Squamocolumnar junction
- SOS – SOS clinic, The Gambia
- STI – Sexually transmitted infection
- TDF – Tenofovir disoproxil fumarate
- TV – Trichomonas vaginalis
- VIA – Visual inspection with acetic acid
- WHO – World Health Organization

INTRODUCTION

- This document describes the standards of care for managing participants who test positive for human papillomavirus (HPV), Neisseria gonorrhoea (NG), Chlamydia trachomatis (CT), Trichomonas vaginalis (TV), Mycoplasma genitalium (MG), syphilis, human immunodeficiency virus (HIV), hepatitis B and hepatitis C during the PHASE survey.
- All participants will receive pre-test counselling during which they will be encouraged to ask any questions they may have until they are satisfied. They will also receive post-test counselling based on their test results.
- Point of care tests will be performed for syphilis, HIV, hepatitis B and hepatitis C and as such, participants will receive their test results on the day of testing, barring any unforeseen circumstances. Medication or referral will generally take place on the day as applicable.
- Laboratory-based tests will be performed for HPV, NG, CT, TV and MG. Participants who test positive for any of these infections will have their results communicated to them by survey staff and medications administered as appropriate. Some cases will be referred to the appropriate health facilities as required.
- Clinical care of survey participants will be conducted in line with best clinical practices as well as standard national and international guidelines.
- Participants who test positive for any of the infections tested for during the survey will be encouraged during counselling to disclose their results to their sexual partner(s) and to encourage them to visit the nearest STI treatment centre for evaluation. The survey staff will not disclose participants' results to their partner(s) or relative(s).
- Participants will only receive treatment for infectious agents mentioned above. Participants will be referred to nearby health facilities to manage any other medical complaints.
- Only PHASE survey participants will receive clinical care from survey staff.

MANAGEMENT OF HIGH-RISK HPV-POSITIVE CASES:

- Participants who test positive for high-risk HPV are expected to receive further assessment and potential treatment. MRCG will work with the SOS clinic to provide such assessment and treatment to survey participants fulfilling the recommended criteria. The SOS clinic has an established cervical cancer screening and treatment service at Bakoteh. Details of the management of high-risk HPV-positive participants are discussed below.
- The approach to the management of high-risk HPV-positive participants will be consistent with the WHO guideline, recommending a screen, triage and treat approach.
- All participants aged 25 years and above, regardless of whether or not they have tested positive for HIV, who have a high-risk HPV type detected in their urine, will be eligible for cervical cancer screening.

  Note: The decision to perform cervical cancer screening for high-risk HPV-positive participants starting at age 25 was guided by our IARC collaborators, along with input from the SOS clinic cervical cancer screening team. The WHO guidelines for managing HPV infection recommend cervical cancer screening for HPV-positive females aged 30 years and above.

- Participants will be eligible for cervical cancer screening based on the IARC classification of HPV types. Specifically, only individuals infected with Group 1 types (HPV 16, 18, 31, 35, 39, 45, 51, 52, 56, 58, 59, and 66) and Group 2A type (HPV 68) will be considered to have high-risk HPV (HR-HPV) types of interest.
- The SOS clinic team will periodically conduct the cervical cancer screening, facilitated by our team, within the district where the participant resides, using visual inspection with acetic acid (VIA), and then thermal ablation of the cervical lesions if indicated.
- Further investigations, such as colposcopy and/or cervical biopsy for histology, will be performed as indicated by the SOS clinic and their collaborators.
- Cases of complicated infections will be referred to the Edward Francis Small Teaching Hospital as required for further management.
- The SOS clinic will be responsible for further follow-up of all participants if required.
- During clinical care visits for the other STIs, the PHASE survey clinical team will provide counselling to all high-risk HPV positive cases who are not eligible for cervical cancer screening per the criteria above. These participants will receive post-test counselling where they will be encouraged to re-test for HPV after 12 months.

Adapted from the WHO guideline for screening and treatment of cervical pre-cancer lesions for cervical cancer prevention, second edition, 2021.

MANAGEMENT OF HEPATITIS B AND C POSITIVE PARTICIPANTS

- All participants testing positive for hepatitis B and/or hepatitis C will be counselled and referred to the MRCG Liver Research Group for further management.
- The MRCG Liver Research Group will periodically set up treatment centres at central locations within the study area where they will attend to all eligible participants. The survey team will facilitate this.
- The participant will not need to pay for this service.

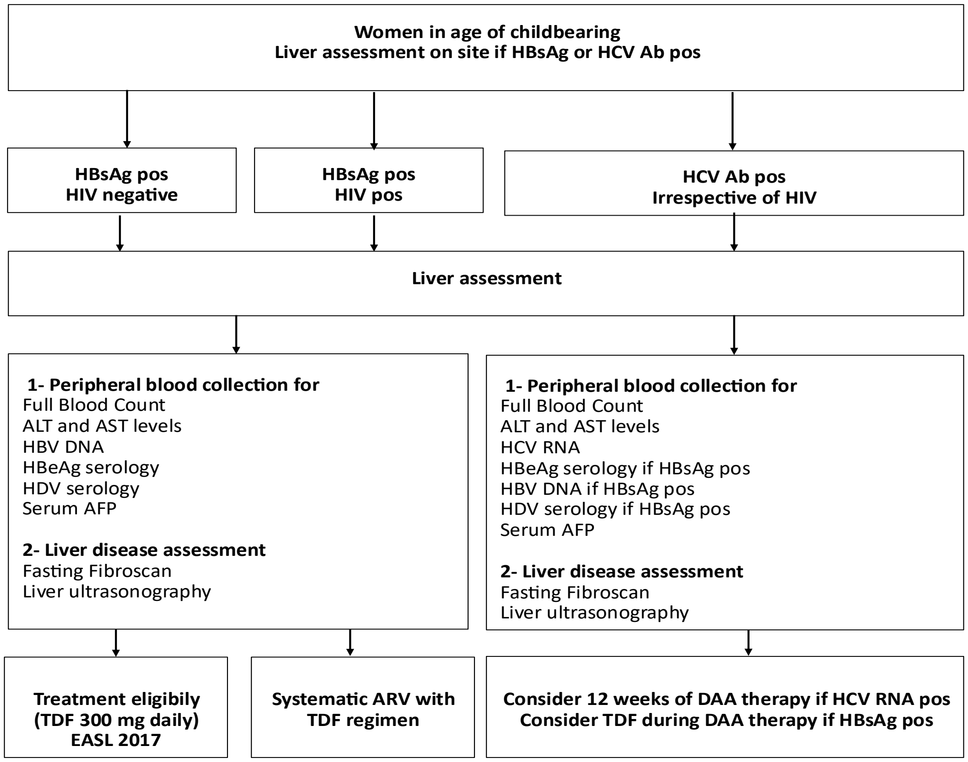

Figure 1. *Algorithm developed by the MRCG Liver Research Group for managing Hepatitis B and Hepatitis C infections. The chart below shows the management protocols used by the*

MRCG Liver Research Group. Point of Care (POC) tests are used initially to screen for hepatitis B and C.

<u>MANAGEMENT OF PARTICIPANTS TESTING POSITIVE FOR SYPHILIS, NEISSERIA GONORRHOEA, CHLAMYDIA TRACHOMATIS, TRICHOMONAS VAGINALIS AND MYCOPLASMA GENITALIUM</u>

Participants who test positive for these infections will have the infections treated by the survey team.

- Participants with clinical manifestations of complicated disease will be referred to the Basse hospital for further management.

INFECTION	TREATMENT	ALTERNATIVE	PREGNANCY
SYPHILIS	IM benzathine penicillin G 2.4 million IU single dose weekly for 3 weeks.	Oral doxycycline 100mg twice daily for 14 days. OR<br><br>Oral erythromycin 500mg 4 times a day for 14 days	•IM benzathine penicillin G 2.4 million IU single dose. OR<br><br>Oral erythromycin 500mg 4 times a day for 14 days
NEISSERIA GONORRHOEA	IM ceftriaxone 250mg single dose plus oral azithromycin 1g single dose.	Oral cefixime 400mg single dose plus oral azithromycin 1g single dose.	IM ceftriaxone 250mg single dose plus oral azithromycin 1g single dose. OR<br><br>Oral cefixime 400mg single dose plus oral azithromycin 1g single dose.
CHLAMYDIA TRACHOMATIS	Oral doxycycline 100mg twice daily for 7 days.	Oral azithromycin 1g single dose. OR<br><br>Oral erythromycin 500mg 4 times daily for 7 days.	Oral erythromycin 500mg 4 times daily for 7 days. OR<br><br>Oral azithromycin 1g single dose.
TRICHOMONAS VAGINALIS	Oral metronidazole 2g single dose. OR<br><br>Oral metronidazole 500mg twice daily for 7 days.	Oral tinidazole 2g single dose. OR<br><br>Oral tinidazole 500mg twice daily for 5 days.	Oral metronidazole 2g single dose.<br><br>Metronidazole gel 0.75% one full applicator (5 grams) intravaginally, twice daily for 7 days.

MYCOPLASMA GENITALIUM	Oral azithromycin 500mg on day 1, followed by 250mg daily on days 2-5.	Oral doxycycline 100mg twice daily for 7 days, followed by oral moxifloxacin 400mg once daily for 7 days.<br>OR<br><br>Oral doxycycline 100mg twice daily for 7 days, followed by a single dose of oral azithromycin 1g followed by 500mg orally daily for 3 additional days (2.5g total).	Oral azithromycin 500mg daily on day 1, followed by 250mg daily on days 2-5.

Table 1: *Regimen for treating selected sexually transmitted infections. Adapted from WHO Guidelines For The Management Of Symptomatic Sexually Transmitted Infections and CDC STI Treatment Guidelines, 2021.*

REFERENCES

1. WHO Guidelines For The Management Of Symptomatic Sexually Transmitted Infections.
2. CDC STI Treatment Guidelines, 2021.

MANAGEMENT OF HIV POSITIVE PARTICIPANTS

- All participants testing positive from the HIV POC test will receive post-test counselling and be referred to the National AIDS Control Programme at the Basse hospital to initiate antiretroviral therapy if not already known to be HIV positive and under care.
- HIV positive participants with co-infections with hepatitis B or C will also be referred to the MRC Liver Research Group for co-management.
- HIV positive participants who also test positive for syphilis will receive syphilis treatment on site as described above from the survey team prior to referral to the National AIDS Control Programme at the Basse hospital.
- HIV positive participants who also test positive for NG, CT, TV or MG will receive treatment for those infections as described above from the survey team.
- Any other sexually transmitted co-infections will be managed as appropriate by the Basse hospital.
- Referrals to the Basse hospital will be done when indicated.

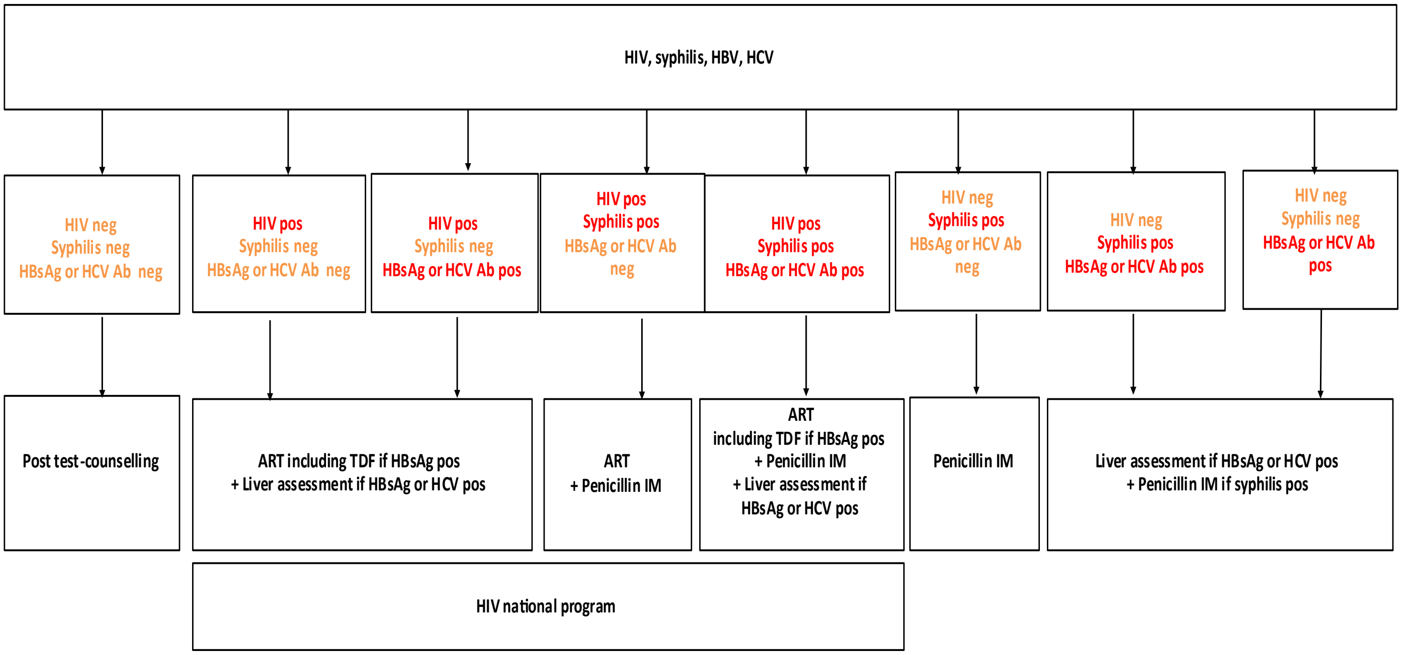

Figure 2. *Algorithm developed by the MRCG Liver Research Group for managing HIV, Syphilis, Hepatitis B and Hepatitis C infections.*

SOS GAMBIA GUIDELINES FOR PERFORMING VISUAL SCREENING

Visual methods are not recommended for use in menopausal women, because their transition zone is most often inside the endocervical canal and not visible on speculum inspection.

Preparation

1. Explain the procedure, how it is done, and what a positive test means.
2. Ensure that the woman has understood and obtain informed consent.
3. Do a speculum examination.

Procedure

1. Adjust the light source in order to get the best view of the cervix.
2. Use a cotton swab to remove any discharge, blood or mucous from the cervix.
3. Identify the squamocolumnar junction (SCJ), and the area around it.
4. Soak a clean swab in 3-5% acetic acid and apply to the cervix; Dispose of swab in leak proof container or dustbin.
5. Wait a minute or two to allow colour changes to develop. Observe the cervix for acetowhite changes. Give special attention to abnormalities close to the transformation zone.

Inspect the SCJ carefully :

a. Check whether the cervix bleeds easily and report it.
b. Look for any raised and thickened white plaques or acetowhite epithelium if you used acetic acid.
c. If in doubt, reapply acid acetic .
d. Remove any blood debris appearing during the inspection.
e. When visual inspection has been completed, use a fresh swab to remove any remaining acetic acid from the cervix and vagina.
f. Gently remove the speculum.

After the procedure

1. Record your observations and test result.
2. Draw a map of any abnormal findings on the record form.
3. Discuss the result of the screening test with the patient.
4. If the test is negative, tell her she should have another test in 10 years.
5. If the test is positive or cancer is suspected, tell her what the next recommended steps are.

6. If she needs to be referred for further testing or treatment, make arrangements and provide her with all necessary forms and instructions before she leaves.
7. If you can make the appointment immediately, do so.

<u>SOS Gambia Guidelines For Treatment Of VIA Positive Lesions By Thermal Ablation</u>

1. Explain the procedure to the woman and document verbal consent.
2. Select appropriate probe.
3. Turn on the thermal ablation machine.
4. Turn the dial fully to the right and wait for the green light to show, this means the probe is at the correct temperature.
5. Apply the probe to the area to be treated, making sure you do not touch the vaginal wall. Leave in place for 30 seconds. If another area is to be treated also reapply for 30 seconds.
6. Remove the probe from the speculum and place in probe holder. Turn off the machine.
7. Remind the woman about watery/ bloody discharge and to abstain from sexual intercourse for 4 weeks. Answer any questions and ask her to return in 6 months.
8. Once the woman has left, remove the used probe, wash thoroughly with a toothbrush in chlorine, rinse in water, dry and return to the machine and auto-sterilise.

*NB:IT IS THE CLINICIAN'S RESPONSIBILITY TO CLEAN AND AUTO-STERILISE THE PROBE

To auto-sterilise:

1. Re-attach probe.
2. Turn on thermal ablator.
3. Press auto-sterilise – orange button will light up.
4. When orange button goes out the auto-sterilise is complete.
5. In some models, select 120^{0}C and heat probe for one minute to sterilise.
6. Turn off the machine.
