# Supplementary material for: Human papillomavirus, sexually transmitted infections, and antimicrobial resistance in West Africa: Estimating population burden and understanding exposures to accelerate vaccine impact and drive new interventions: The PHASE survey protocol
Source: PLoS One. 2025 Sep 22;20(9):e0332842. doi: 10.1371/journal.pone.0332842 (PMC12453253; doi:10.1371/journal.pone.0332842)
Supplement: S4 Appendix — (PDF) [file pone.0332842.s004.pdf]

International HPV Reference Center
Sweden

Professor Joakim Dillner

EQUALIS

Global HPV DNA genotyping Proficiency Study 2024

Bruno Pichon
Medical Research Council Unit of The Gambia
At the London School of Hygien
Atlandic Boulevard, PO box 273
0000 Fajara
The Gambia

We appreciate your participation in the Global HPV genotyping Proficiency Study.

Herewith, we enclose the results of your laboratory in the testing of the 2024 Global HPV DNA proficiency panel.

A test is regarded as proficient in typing if it can detect 50 International Units (IU) / 5 µl of HPV 16 and HPV 18 DNA, and 500 genome equivalents (GE) / 5 µl of the other HPV types included in the panel both in samples with single and multiple plasmids.
In addition, the specificity of the reported types should be 100 % (no false positive result).

Accordingly, your data set using the Seegene Allplex HPV 28 genotyping assay is proficient for detection of HPV 6, 11, 16, 18, 31, 33, 35, 39, 45, 51, 52, 56, 58, 59, 68a and 68b.

Should you have any technical questions, please contact Dr Joakim Dillner at joakim.dillner@ki.se.
An overall summary report of this proficiency study will be prepared and sent to you in due course.

Sincerely Yours

Joakim Dillner

International HPV Reference Center, Karolinska Institutet,

Karolinska University Hospital Huddinge, 141 86 Stockholm, Sweden

Visiting address Division of Pathology, F56

Phone +46 8 58581168 Fax +46 8 58587730 E-mail Joakim.Dillner@ki.se

Reg. nr.: SE202100297301

International HPV Reference Center
Sweden

Professor Joakim Dillner

Annex 1: Your results in relation to the contents of the proficiency panel.

Panel ID	HPV type(s) in the panel	Content (IU or GE per 5 µl)	Your results Allplex HPV 28 5 µl input volume
1	45	50	45
2	11	50	11
3	68b	50	68
4	16, 35, 51, 56	50	16, 35, 51, 56
5	59	50	59
6	51	50	51
7	39	500	39
8	18	5	18
9	6, 18, 31, 59	50	6, 18, 31, 59
10	16	50	16
11	33	50	33
12	56	500	56
13	6, 18, 31, 59	500	6, 18, 31, 59
14	Neg	0	none
15	51	500	51
16	58	500	58
17	68a	50	68
18	16, 35, 51, 56	500	16, 35, 51, 56
19	6	50	6
20	33	500	33
21	59	500	59
22	11	500	11
23	11, 33, 45, 68b	500	11, 33, 45, 68
24	39	50	39
25	56	50	56
26	39, 52, 58, 68a	500	39, 52, 58, 68
27	16	5	16
28	35	500	35
29	52	50	52
30	68a	500	68
31	11, 33, 45, 68b	50	11, 33, 45, 68
32	39, 52, 58, 68a	50	39, 52, 58, 68
33	18	50	18
34	31	50	31
35	52	500	52

International HPV Reference Center, Karolinska Institutet,

Karolinska University Hospital Huddinge, 141 86 Stockholm, Sweden

Visiting address Division of Pathology, F56

Phone +46 8 58581168 Fax +46 8 58587730 E-mail Joakim.Dillner@ki.se

Reg. nr.: SE202100297301

36	35	50	35
37	6	500	6
38	31	500	31
39	45	500	45
40	68b	500	68
41	58	50	58
A	16	25	16
B	16	2500	16
C	Negative	0	none

International HPV Reference Center, Karolinska Institutet,

Karolinska University Hospital Huddinge, 141 86 Stockholm, Sweden

Visiting address Division of Pathology, F56

Phone +46 8 58581168 Fax +46 8 58587730 E-mail Joakim.Dillner@ki.se

Reg. nr.: SE202100297301
