# Supplementary material for: Human papillomavirus, sexually transmitted infections, and antimicrobial resistance in West Africa: Estimating population burden and understanding exposures to accelerate vaccine impact and drive new interventions: The PHASE survey protocol
Source: PLoS One. 2025 Sep 22;20(9):e0332842. doi: 10.1371/journal.pone.0332842 (PMC12453253; doi:10.1371/journal.pone.0332842)
Supplement: S5 Appendix — (PDF) [file pone.0332842.s005.pdf]

The Gambia Government/MRC Joint
ETHICS COMMITTEE

[illegible]

Professor Ed Clarke
MRCG at LSHTM

7 November 2023

Dear Professor Clarke

Study Title: HPV, sexually transmitted infections, and antimicrobial resistance in West Africa: estimating population burden and understanding exposures to accelerate vaccine impact and drive new interventions

Project ID/ethics ref: 29840

Thank you for submitting your application which was considered by the Gambia Government/MRCG Joint Ethics Committee at its meeting held on 26 October 2023

Confirmation of ethical opinion

On behalf of the Committee, I am pleased to confirm a favourable ethical opinion for the above research on the basis described in the application form, protocol and supporting documentation.

Approved documents

The final list of documents reviewed and approved by the Committee is as follows:

Document Type	File Name	Date	Version
Other	Larry Kotei_GCP	21/01/2021	Not applicable
Other	SOP-CTS-015_CT-Informed consent _intranet (1)	22/09/2021	6.0
Other	Ousubie Jawla_GCP	21/01/2022	Not applicable
Other	Adedapo Bashorun_GCP	05/05/2022	Not applicable
Other	Adedapo Bashorun_Research Ethics	06/05/2022	Not applicable
Other	Ed Clarke_GCP	12/02/2023	Not applicable
Investigator CV	Ousubie Jawla_30May2023	30/05/2023	Not applicable
Investigator CV	Larry Kotei_20Sep2023	20/09/2023	Not applicable
Investigator CV	Ed Clarke_23Sep2023	23/09/2023	Not applicable
Investigator CV	Adedapo Olufemi Bashorun_23Sep2023	23/09/2023	Not applicable
Protocol / Proposal	Sociodemographic_V1.0_23Sep2023	23/09/2023	1.0
Protocol / Proposal	ACASI_V1.0_23Sep2023	23/09/2023	1.0
Protocol / Proposal	STI symptoms_V1.0_23Sep2023	23/09/2023	1.0
Protocol / Proposal	231008 - PHASE Survey_Protocol_V1.1_08Oct2023_tracked	08/10/2023	1.1
Protocol / Proposal	231011 - PHASE Survey_Protocol_V1.1_08Oct2023_clean	08/10/2023	1.1
Information Sheet	231011_ICD_Adults_PHASE Survey_v1.1_08Oct2023_tracked	08/10/2023	1.1
Information Sheet	231011_ICD_Adults_PHASE Survey_v1.1_08Oct2023_clean	08/10/2023	1.1
Information Sheet	231011_ICD_Parent_PHASE Survey_v1.1_08Oct2023_tracked	08/10/2023	1.1
Information Sheet	231011_ICD_Parent_PHASE Survey_v1.1_08Oct2023_clean	08/10/2023	1.1
Information Sheet	231011_Assent_15-17-years_PHASE Survey_v1.1_08Oct2023_tracked	08/10/2023	1.1
Information Sheet	231011_Assent_15-17-years_PHASE Survey_v1.1_08Oct2023_clean	08/10/2023	1.1
Information Sheet	231011_ICD_SubStudy_Male_PHASE Survey_v1.1_08Oct2023_tracked	08/10/2023	1.1
Information Sheet	231011_ICD_SubStudy_Male_PHASE Survey_v1.1_08Oct2023_clean	08/10/2023	1.1
Information Sheet	231011_ICD_SubStudy_Female_PHASE Survey_v1.1_08Oct2023_tracked	08/10/2023	1.1
Information Sheet	231011_ICD_SubStudy_Female_PHASE Survey_v1.1_08Oct2023_clean	08/10/2023	1.1
Covering Letter	Response to SCC_LEO29840	11/10/2023	Not applicable

After ethical review

The Principal Investigator (PI) or delegate is responsible for informing the Ethics Committee of any subsequent changes to the application. These must be submitted to the Committee for review using an Amendment Form. Amendments must not be initiated before receipt of written favourable opinion from the Committee.

The PI or delegate is also required to notify the Ethics Committee of any protocol violations and/or Suspected Unexpected Serious Adverse Reactions (SUSARs) which occur during the project by submitting a Serious Adverse Event form. An annual report should be submitted to the Committee using an Annual Report Form on the anniversary of the approval of the study during the lifetime of the study. At the end of the study, the PI or delegate must notify the Committee using an End of Study Form.

All aforementioned forms are available on the ethics online applications website and can only be submitted to the committee via the website at: http://leo.lshtm.ac.uk. Additional information is available at: www.lshtm.ac.uk/ethics.

With best wishes

Yours sincerely

Dr Mohammadou Kabir Cham

Chairperson, Gambia Government/MRCG Joint Ethics Committee

C/O MRC Unit The Gambia at LSHTM
PO Box 273 Banjul, The Gambia
West Africa
Switchboard (+220) 4495442/6 Ext 2308
Fax (+220) 4495919/4496513
E-mail: ethics@mrc.gm
Intranet: http://mrcpo[illegible]_Committees/SCC/SitePages/Home.aspx
Webpage:https //mrcportal.mrc.gm/Committees/SCC/SitePages/Home.aspx

London School of Hygiene & Tropical Medicine
Keppel Street, London WC1E 7HT
United Kingdom
Switchboard: +44 (0)20 7636 8636

www.lshtm.ac.uk

LONDON SCHOOL of HYGIENE &TROPICAL MEDICINE

Observational / Interventions Research Ethics Committee

Professor Ed Clarke

LSHTM

21 November 2023

Dear Dr Clarke

Study Title: HPV, sexually transmitted infections, and antimicrobial resistance in West Africa: estimating population burden and understanding exposures to accelerate vaccine impact and drive new interventions.

LSHTM Ethics Ref: 29840

Thank you for your application for the above research project which has now been considered by the Observational Committee via Chair's Action.

Confirmation of ethical opinion

On behalf of the Committee, I am pleased to confirm a favourable ethical opinion for the above research on the basis described in the application form, protocol and supporting documentation, subject to the conditions specified below.

Conditions of the favourable opinion

Approval is dependent on local ethical approval having been received, where relevant.

Approved documents

The final list of documents reviewed and approved is as follows:

Document Type	File Name	Date	Version
Other	Larry Kotei_GCP	21/01/2021	Not applicable
Other	SOP-CTS-015_CT-Informed consent _intranet (1)	22/09/2021	6.0
Other	Ousubie Jawla_GCP	21/01/2022	Not applicable
Other	Adedapo Bashorun_GCP	05/05/2022	Not applicable
Other	Adedapo Bashorun_Research Ethics	06/05/2022	Not applicable
Other	Ed Clarke_GCP	12/02/2023	Not applicable
Investigator CV	Ousubie Jawla_30May2023	30/05/2023	Not applicable
Investigator CV	Larry Kotei_20Sep2023	20/09/2023	Not applicable
Investigator CV	Ed Clarke_23Sep2023	23/09/2023	Not applicable
Investigator CV	Adedapo Olufemi Bashorun_23Sep2023	23/09/2023	Not applicable
Protocol / Proposal	Sociodemographic_V1.0_23Sep2023	23/09/2023	1.0
Protocol / Proposal	ACASI_V1.0_23Sep2023	23/09/2023	1.0
Protocol / Proposal	STI symptoms_V1.0_23Sep2023	23/09/2023	1.0
Protocol / Proposal	231008 - PHASE Survey_Protocol_V1.1_08Oct2023_tracked	08/10/2023	1.1
Protocol / Proposal	231011 - PHASE Survey_Protocol_V1.1_08Oct2023_clean	08/10/2023	1.1
Information Sheet	231011_ICD_Adults_PHASE Survey_v1.1_08Oct2023_tracked	08/10/2023	1.1
Information Sheet	231011_ICD_Adults_PHASE Survey_v1.1_08Oct2023_clean	08/10/2023	1.1
Information Sheet	231011_ICD_Parent_PHASE Survey_v1.1_08Oct2023_tracked	08/10/2023	1.1

Document Type	File Name	Date	Version
Information Sheet	231011_ICD_Parent_PHASE Survey_v1.1_08Oct2023_clean	08/10/2023	1.1
Information Sheet	231011_Assent_15-17-years_PHASE Survey_v1.1_08Oct2023_tracked	08/10/2023	1.1
Information Sheet	231011_Assent_15-17-years_PHASE Survey_v1.1_08Oct2023_clean	08/10/2023	1.1
Information Sheet	231011_ICD_SubStudy_Male_PHASE Survey_v1.1_08Oct2023_tracked	08/10/2023	1.1
Information Sheet	231011_ICD_SubStudy_Male_PHASE Survey_v1.1_08Oct2023_clean	08/10/2023	1.1
Information Sheet	231011_ICD_SubStudy_Female_PHASE Survey_v1.1_08Oct2023_tracked	08/10/2023	1.1
Information Sheet	231011_ICD_SubStudy_Female_PHASE Survey_v1.1_08Oct2023_clean	08/10/2023	1.1
Covering Letter	Response to SCC_LEO29840	11/10/2023	Not applicable

After ethical review

The Chief Investigator (CI) or delegate is responsible for informing the ethics committee of any subsequent changes to the application. These must be submitted to the committee for review using an Amendment form. Amendments must not be initiated before receipt of written favourable opinion from the committee.

The CI or delegate is also required to notify the ethics committee of any protocol violations and/or Suspected Unexpected Serious Adverse Reactions (SUSARs) which occur during the project by submitting a Serious Adverse Event form.

An annual report should be submitted to the committee using an Annual Report form on the anniversary of the approval of the study during the lifetime of the study. The date the first annual report is due is 21/11/2024

At the end of the study, the CI or delegate must notify the committee using the End of Study form.

All aforementioned forms are available on the ethics online applications website and can only be submitted to the committee via the website at: http://leo.lshtm.ac.uk.

Further information is available at: www.lshtm.ac.uk/ethics.

Yours sincerely,

Professor David Leon and Professor Clare Gilbert

Co-Chairs

ethics@lshtm.ac.uk
http://www.lshtm.ac.uk/ethics/

Improving health worldwide
